# Photobiomodulation in post menopause genitourinary syndrome—Study protocol for a randomized, double-blind, controlled clinical protocol

**DOI:** 10.1371/journal.pone.0313324

**Published:** 2024-12-02

**Authors:** Silvia Regina dos Santos Pereira, Raquel Agnelli Mesquita-Ferrari, Luciana Toledo Costa Salviatto, Cícero Dayves da Silva Bezerra, Vanessa Dalapria, Erika da Silva Mello, Luciana Almeida-Lopes, Paulo Sérgio Bossini, Marcela Leticia Leal Gonçalves, Alessandro Melo Deana

**Affiliations:** 1 Post Graduation Program in Biophotonics Medicine, Universidade Nove de Julho, São Paulo, SP, Brazil; 2 Institute of Research and Education in the Health Area (NUPEN), São Carlos, SP, Brazil; 3 Post Graduation Program in Health and Environment, Universidade Metropolitana de Santos–UNIMES, Santos, SP, Brazil; Universidade dos Açores Departamento de Biologia: Universidade dos Acores Departamento de Biologia, PORTUGAL

## Abstract

**Introduction:**

Genitourinary Syndrome of Menopause (GSM) defines a set of symptoms associated with an estrogen deficit involving alterations in organs genitourinary and that results in several urinary, genital, and sexual alterations. Brazilian women live about a third of their life after menopause, where hormonal changes occur along with clinical manifestations, characterized by vaginal and vulvar dryness, burning sensation, discomfort, vulvovaginal irritation, lack of lubrication, dyspareunia and urinary incontinence. Fractionated photothermolysis and radiofrequency systems, alone or in combination were tested to improve GSM.

**Objective:**

The goal of this study is to elaborate a protocol to evaluate the clinical response of patients with symptoms of GSM after the application of photobiomodulation in the vulvar region.

**Method:**

In this randomized, double-blind, placebo-controlled study protocol, women over 50 years of age who are in the postmenopausal period (amenorrhea for at least 12 months, with no pathology involved) with one or more symptoms of GSM will be randomly divided into two groups. The treatment group (n = 30) will receive four consecutive applications, weekly, using DMC laser diode (λ = 808 nm), 4J per point, 100mW of power, 1,016W/cm^2^, 8 sites in the vulvar region, The Placebo Group (n = 30) will be handled as treated, but with the laser turned off. The quality of life will be assessed using female sexual functioning index (FSFI-6), urinary incontinence questionnaire (ICIQ-SF), Quality of life will be analyzed using the female sexual functioning index (FSFI-6). The intensity of menopausal symptoms will be evaluated using a visual analogue scale (VAS), the vulvo vaginal atrophy will be measured by the Vaginal Health Index (VHI). Also, the vaginal temperature will be measured using a thermal camera, the pressure of the pelvic floor force (vaginal dynamometer) and a 1-hour Pad Test will be performed to quantify the urinary loss. With this procedure, we intend to obtain an overall better life quality and diminished symptoms in women with GSM. All assessments will be performed prior to the first irradiation and after the last one.

**Trial registration:**

This protocol is registered at ClinicalTrials.gov under the number NCT05557799.

## Introduction

### Study design

**Fig 1:**
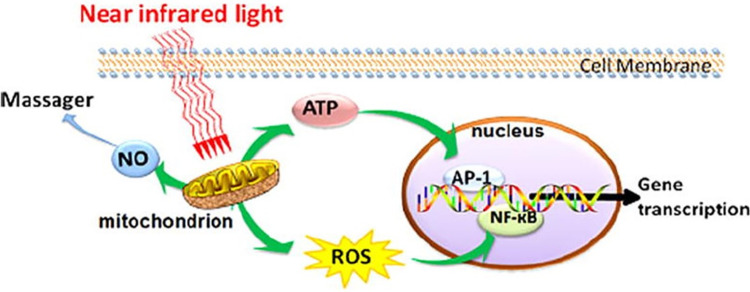
Cellular mechanisms of PBM.

This study protocol was designed as a prospective, randomized, double-blind, controlled trial according to the 2023 SPIRIT (Standard Protocol Items: Recommendations for Interventional Trials) Statement and SPIRIT (Fig 3) and it will be conducted at Nove de Julho University and the Municipal Health Department of Vargem Grande Paulista, SP in 2023–2024. Fig 1 shows the SPIRIT statement. All participants were required to sign an informed written consent. The recruitment will be held from February 01^st^ 2024 to June 30^rd^ 2024

### Contextualization

Genitourinary Syndrome of Menopause (GSM) refers to a set of symptoms and signs associated with estrogen deficiency, characterized by changes in the genitourinary organs resulting in various urinary, genital, and sexual alterations [1].

GSM or its characteristics manifest in approximately 15% of women in the premenopausal stage and 40–54% of women in the postmenopausal stage [2, 3]. In the United States, GSM is estimated to affect 8% to 22% of women in the premenopausal stage and 40% to 57% of women in the postmenopausal stage. Surprisingly, only 1 in 5 women experiencing GSM-related symptoms seek medical attention [4]. International literature indicates that only 59% of these women seek medical help, whereas in Europe, only 4% of women report vulvovaginal symptoms. About 25% of women with GSM seek medical care, and only 54% discuss their sexual health with healthcare professionals when asked, while 33% choose not to address this issue during a consultation [5].

In Brazil, studies show that only 59% of women experiencing symptoms related to urogenital atrophy seek medical help. The complaint of vaginal dryness is present in 34.9% of women, and respondents reported that the climacteric negatively impacts their sexuality. In the United States, 59% of women reported sexual dissatisfaction [6–8].

Among the observed signs and symptoms, studies found a high frequency of complaints such as dyspareunia (44%), irritation (37%), vaginal dryness (55%), recurrent urinary tract infections (5% to 17%), and urge incontinence in women above 60 years (15% to 35%). The prevalence of atrophic vaginitis was 4%, increasing to 25% in the first year after menopause and 47% after three years. Note that dyspareunia leads to a reduction in the frequency of sexual intercourse, with 72% of women having intercourse only once a month and 34% weekly [9, 10].

The complaint of chronic incontinence is one of the main signs and symptoms of genitourinary syndrome in postmenopausal women. Studies point out that the loss of conscious and voluntary bladder control is a common problem that can affect anyone, but its prevalence increases in the female sex and with advancing age [11]. With the increase in women’s life expectancy, urinary incontinence must be considered a concern by healthcare professionals, directly affecting healthy aging and quality of life in this age group. About 10% of women visiting the gynecologist list urine loss as their main complaint, which represents a negative impact on quality of life [12–14].

This condition is often treated with physiotherapy, which aims to educate about urinary function, utilize pelvic floor muscle (PFM) function, and strengthen these muscles through techniques and exercises [15]. Common interventions in physiotherapy include kinesiotherapy (with or without vaginal cones), electrostimulation, biofeedback, behavioral therapy, and body awareness within primary and secondary healthcare services [16].

Vaginitis is also a common complaint in GSM, and gynecologists usually recommend hormonal and non-hormonal treatments to address it. Non-hormonal lubricants and moisturizers can be helpful for mild to moderate vaginal dryness, but they might interfere with sexual spontaneity and be impractical in application, leading to low long-term adherence. On the other hand, topical estrogen application is considered the "gold standard" treatment for moderate to severe cases or those not responsive to non-hormonal treatments [17].

However, topical estrogen application has limitations, such as: lack of long-lasting action, high recurrence rate after discontinuation, and action limited to the vaginal epithelial surface without effects on extracellular matrix renewal and vascularization. Additionally, many women discontinue treatment due to the risk of developing hormone-dependent neoplasms in the long term [18, 19].

Alternatively, various types of treatments using a photothermal approach with CO2 lasers, fractional lasers, Er:YAG lasers, and radiofrequency systems, either individually or in combined therapy, have shown improvement in GSM symptoms, promoting morphological changes in vaginal tissues and increasing vaginal blood circulation. Patients report improvement in vaginal dryness, dyspareunia, and stress urinary incontinence [20]. However, the high cost of this method as well as its side effects such as pain and erythema may limit its use in public healthcare [21].

Alternatively, photobiomodulation therapy (PBM) is a different approach that is practical, safe, painless, well-tolerated, without immediate or delayed complications, and can be applied on an outpatient basis for the treatment of GSM. This is particularly relevant for women with atrophic vaginitis (AV) and a history of hormone-dependent neoplasia, for whom estrogen therapy is contraindicated [22].

Photobiomodulation therapy utilizes light at appropriate radiometric parameters that triggers a cascade of cellular effects that result in alterations in local tissues. The primary mechanism proposed to explain the effects of red and infrared light involves modifications in mitochondrial activity, especially in the enzyme cytochrome C oxidase (COX), present in damaged or hypoxic cells [23].

The biochemical mechanism underlying the therapeutic effects PBM is not fully elucidated, nevertheless there is robust evidence indicating that it acts on mitochondria to stimulate adenosine triphosphate production, modulate reactive oxygen species (ROS), and induce transcription factors. Several of these transcription factors are regulated by changes in cellular redox state. These include redox factor-1 (Ref-1), activator protein-1 (AP-1) (a c-Fos and c-Jun complex), nuclear factor kappa B (NF-κB), p53, cyclic AMP response element-binding protein (ATF/CREB), hypoxia-inducible factor (HIF)-1, and HIF-like factor [24, 25]. These transcription factors then trigger protein synthesis, resulting in subsequent effects such as increased cell proliferation and migration, modulation of cytokine levels, growth factors, inflammatory mediators, and enhancement of tissue oxygenation [26].

**Fig 2:**
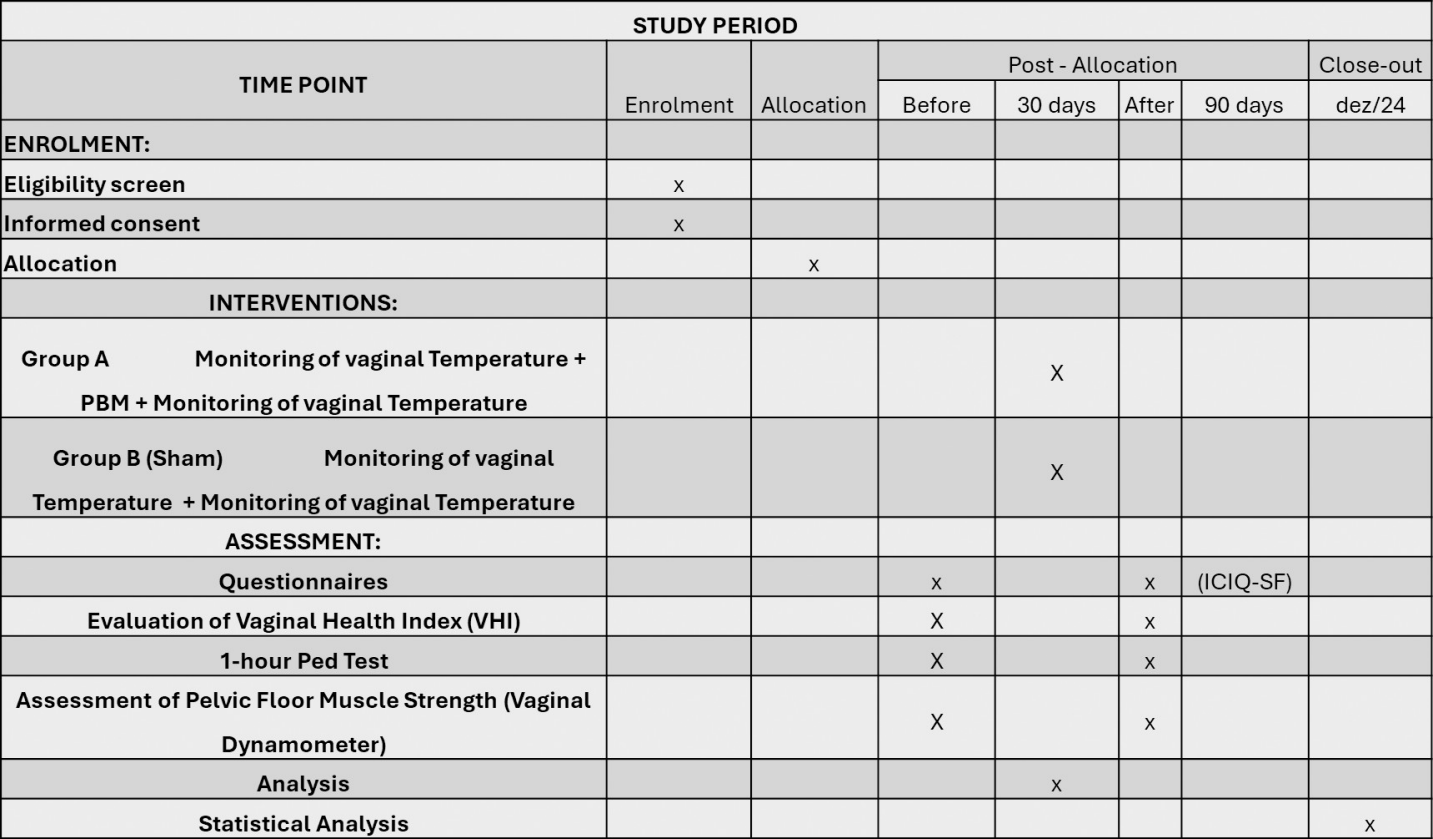
SPIRIT figure for the schedule of enrolment as recommended by 2013 SPIRIT statement.

Fig 2 illustrates the mechanism of action of PBM at the cellular and molecular levels.

Immune cells, such as mast cells, are strongly influenced by PBM, where specific wavelengths of light are capable of triggering mast cell degranulation. PBM also enhances the proliferation, maturation, and motility of fibroblasts, leading to increased production of basic fibroblast growth factor, thereby promoting faster tissue healing [27]. In addition, it can induce vasodilation by triggering relaxation of smooth muscle associated with the endothelium, which is highly relevant for inflammation treatment. This vasodilation enhances oxygen availability to treated cells and allows for increased immune cell trafficking into tissues. These two effects contribute to accelerated healing [28].

Given the current advancements and limitations in the field, there is a significant need for innovative therapeutic strategies to manage Genitourinary Syndrome of Menopause (GSM) in women experiencing urinary incontinence. Therefore, the objective of this study is to evaluate the effects of photobiomodulation on clinical outcomes in postmenopausal women presenting with GSM and urinary incontinence, utilizing a randomized, placebo-controlled clinical trial design.

To our knowledge, this study is the first protocol to investigate photobiomodulation therapy in patients with Genitourinary Syndrome of Menopause.

## Methods

### Ethics

This study is a placebo-controlled, randomized, and double-blind clinical protocol, approved by the Research Ethics Committee of Nove de Julho University (Uninove) number 5.628.333 and approved by the Municipal Health Department of Vargem Grande Paulista, SP.

### Sample size and participants

**Fig 3:**
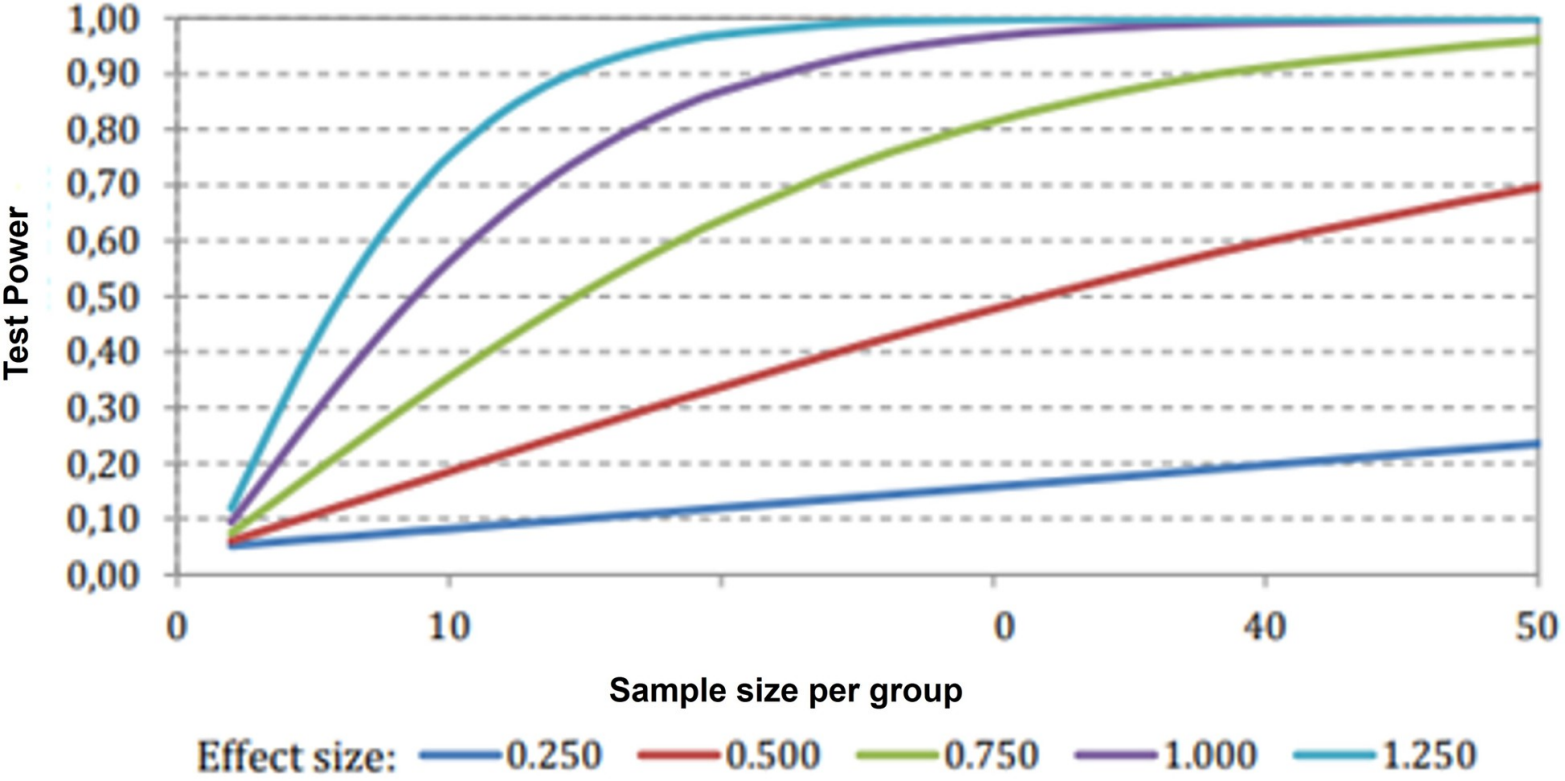
Test power analysis as a function of effect size and sample size.

Since the literature lacks studies on the effects of the photobiomodulaton on GSM, the sample size was calculated using the formula described using the estimated effect size, as described by Kadam and Bhalerao [29]. Considering a type I error of 0.05 and a test power of 80%, the calculated sample size was 60 participants, with 30 in each group (A and B). The Fig 3 shows that, for medium (0.750) and large effects (1.000–1.250), a minimum of 30 patients per group is sufficient to control statistical variance, ensuring a test power greater than 0.80. Considering a 10% of sample loss, a total of 66 patients will be recruited for the study. The Fig 3 shows a plot of the test power as a function of the sample size and estimated effect size.

#### Recruitment

Recruitment will be conducted through the public health system of Vargem Grande Paulista, SP, Brazil. Additionally, recruitment efforts were carried out through the municipality’s Senior Center, social media, and referrals from participants who had previously undergone the project.

### Inclusion and exclusion criteria

#### Inclusion

- Women over 50 years old in the postmenopausal period (amenorrhea for at least 12 months without involved pathology);
- Presenting one or more symptoms of GSM (such as dyspareunia, dryness, irritation, vaginal burning and/or discomfort, vaginal and vulvar atrophy, vaginal dryness, dysuria, polyuria, recurrent urinary infections);
- With complaints of stress and/or urgency urinary incontinence;
- Presenting a normal cervical cytology (Pap smear) performed within the last year.

#### Exclusion

- Woman on hormonal medication for menopausal symptoms in the last 6 months;
- Participants who explicitly declined to participate;
- Participants who had a history of bilateral oophorectomy;
- Subjects with conditions such as:
  ○ recent Acute Myocardial Infarction (AMI);
  ○ neoplasms;
  ○ history of thrombosis;
  ○ liver insufficiency;
  ○ uncontrolled genital bleeding;
  ○ genital condylomatosis;
  ○ active genital herpes.
- Subjects with surgeries of the lower genital tract that would preclude treatment;
- Subjects with photosensitivity.

### Strategies to improve adherence of the participants to the protocol

The participants will be included in a phone message group through which they will be reminded of the follow-up appointments by the researchers.

### Randomization

The participants were randomly assigned to two groups, Group A (treatment) and Group B (placebo), in block-randomization fashion, with each block comprising four participants. After participant selection and assessment, sealed brown envelopes were randomly assigned to each subject containing the treatment allocation until the block was over, afterwards a new block of four allocation (two in each group) was made.

### Blinding

Both the person evaluating the VHI and the participants will not be informed about the group assignments. This ensures that neither the evaluator nor the participants know which group the participants are in (blinding).

### Outcomes

#### Questionnaires

A questionnaire will be administered to the participants during their initial outpatient consultation conducted by the researcher to gather sociodemographic information.

To assess quality of life and sexual satisfaction, validated questionnaires in Brazil will be employed. The Female Sexual Function Index (FSFI) is a tool used to evaluate female sexual response across six domains: desire, arousal, lubrication, orgasm, satisfaction, and pain [30, 31].

The International Consultation on Incontinence Questionnaire—Short Form (ICIQ-SF) is a questionnaire designed to assess the impact of Urinary Incontinence (UI) on quality of life and quantify urinary loss in both male and female patients [32].

The methods for evaluating Genitourinary Syndrome of Menopause (GSM), which describe the participants’ perception of clinical symptoms before and after the proposed treatment, include the Vaginal Health Index Score (VHI). This method encompasses a clinical assessment during a speculum examination, covering five parameters: elasticity, pH, mucosal appearance, moisture, and the presence of vaginal secretion. A qualified healthcare professional will conduct this evaluation during the physical examination, allowing for the diagnosis of Vulvovaginal Atrophy (VVA) [33].

Vaginal pH evaluation will be performed using a pH indicator strip inserted into the middle third of the vagina, in contact with the mucosa, causing a color change in the strip. The nitrazine strip should remain in contact with the vaginal wall for 5 seconds before checking the pH on the colorimetric scale provided by the manufacturer [34].

The Visual Analog Scale (VAS) will be applied as an instrument for assessing the intensity of pain reported by the participants, aiming to monitor the progression of GSM symptoms during treatment. This assessment will be conducted for each of the symptoms, such as dyspareunia, dryness, irritation/burning, itching, and cytorrhagia, both before and after treatment [35].

#### Vulvar temperature

The vulvar temperature measured in degrees Celsius, will be evaluated at the same eight points that were irradiated in the vulvar region using a digital infrared thermometer, allowing for non-contact measurements. The average of the eight readings will be calculated before and after each of the four consecutive weekly sessions of diode laser treatment.

#### Pelvic pressure assessment

To assess the strength of the pelvic floor muscles, we used a biofeedback device that measures pressure in millimeters of mercury (mmHg) through an inflated vaginal probe. This information was displayed analogically on a manometer. The evaluation took place with the participant in a gynecological position, exposing areas of the abdomen, pelvic floor, and legs, enabling a more precise analysis of pelvic muscle contraction [36].

To insert the vaginal probe of the dynamometer, an unlubricated male condom was used to cover it, as sterilization is not feasible. A water-based lubricating gel was applied to the external part of the male condom to ease insertion for women with insufficient vaginal lubrication. The pressure sensor was inserted into the vaginal canal and resting vaginal pressure (muscle relaxation) was measured. The device was calibrated and adjusted, and the participant was instructed to contract her pelvic floor muscles as strongly as possible three times, maintaining the contraction for at least five seconds (maximum vaginal pressure), with a 30-second interval between each contraction. The minimum vaginal pressure was measured during relaxation of the contractions [37].

#### Urinary loss assessment

The Pad Test, also known as the 1-hour pad test, is an effective method for assessing urinary leakage and categorizing Stress Urinary Incontinence (SUI) into mild, moderate, and severe levels. This method is validated by the Standardization Committee of the International Continence Society (ICS) and allows for the measurement of lost urine by weighing the absorbent pad [11].

To perform the Pad Test, the participant should place a pre-weighed absorbent pad near the external urethral opening and drink 500 mL of water, remaining at rest for 15 minutes. Then, she performs activities simulating daily actions such as walking, sitting and standing up ten times, coughing ten times, picking up objects from the floor five times, jogging in place for a minute, and washing hands under running water for a minute. After completing these activities, the absorbent pad is removed and weighed on a precision scale with a minimum reading of 0.1 mg and a maximum capacity of 220 g. Urinary losses are classified as insignificant (up to 1 g), mild (from 1.1 to 9.9 g), moderate (from 10 to 49.9 g), and severe (above 50 g) [38].

### Statistical analysis

The data will be tested for normality using the Shapiro-Wilk test, and those with parametric distribution will be represented by their respective means and standard deviations. In cases where the assumption of parametric distribution is rejected, medians and quartiles will be used. To assess the association between the studied groups and the independent variables, Student’s t-test or Wilcoxon-Mann-Whitney test will be used for parametric and non-parametric data, respectively. All tests will be two-tailed, and the adopted significance level is α = 0.05. The results will be organized in excel spreadsheet and the inferential analysis will be conducted using R.

### Interventions

#### Research flow

**Fig 4:**
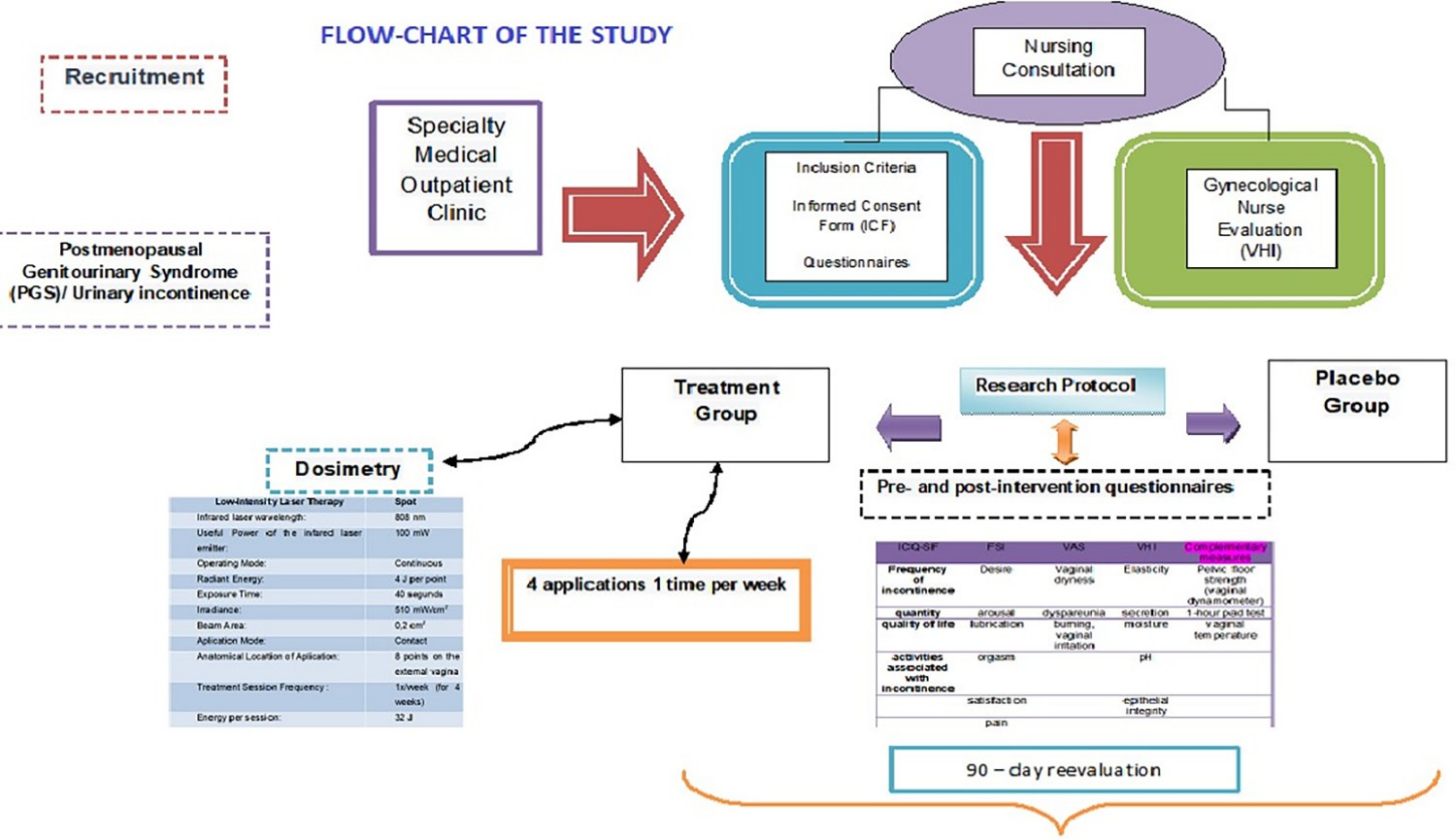
Flow-chart of the study.

Fig 4 Shows the flow-chart of the research.

After the recruitment the subjects a gynecologic analysis and those who fulfill the inclusion criteria performed the first pelvic pressure and urinary loss measurements. After this they were randomly assigned to one of the study groups: sham or photobiomodulation.

#### Photobiomodulation protocol

**Fig 5:**
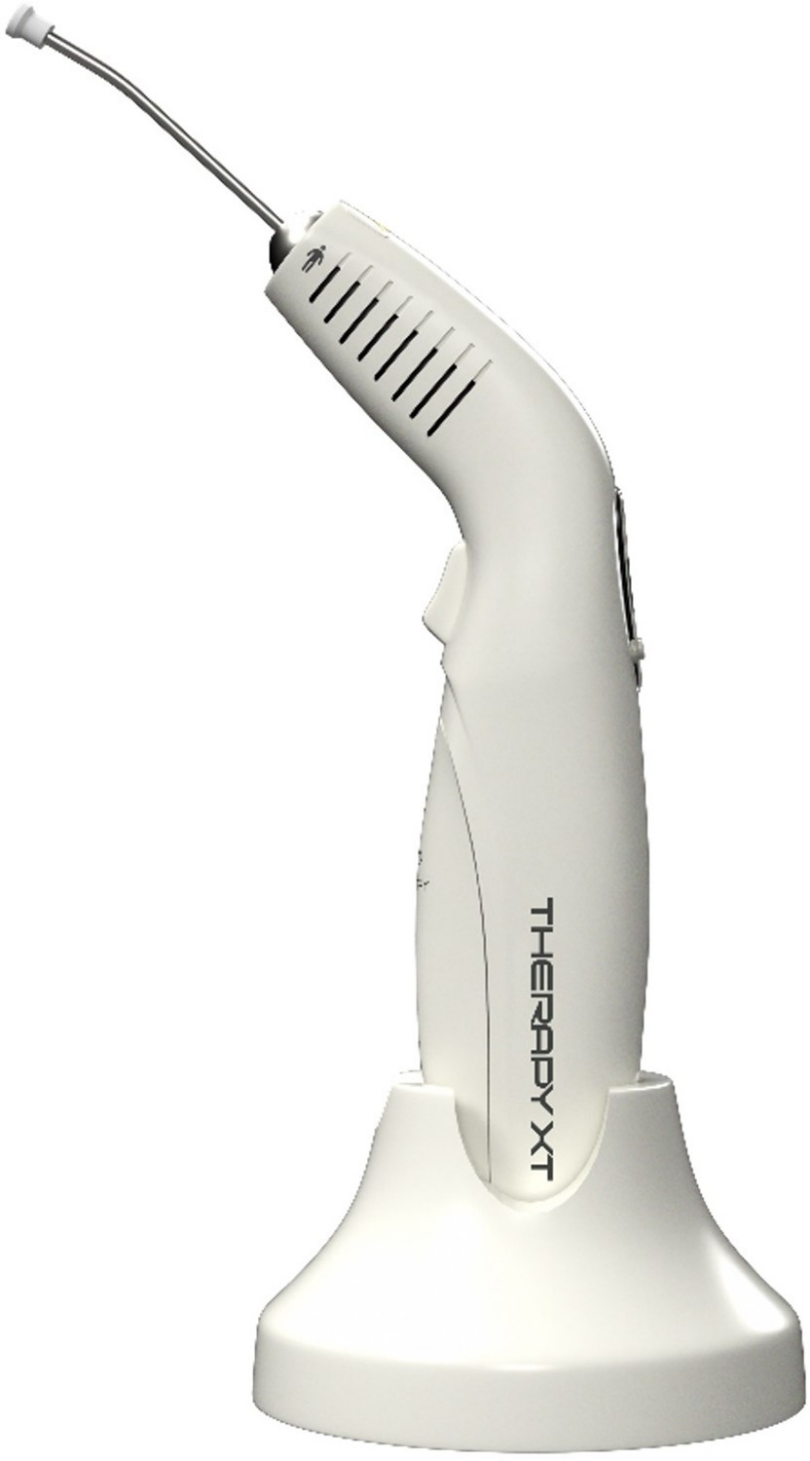
Laser system used in the study (Therapy XT model—DMC).

The photobiomodulation cohort was subjected to the prescribed irradiation regimen in the subsequent manner. Each participating individual will undergo a standardized treatment protocol, involving a one irradiation session per week administered over a span of four consecutive weeks. During each individual session 4 J of radiant energy per application site will be imparted. This energy will be emitted at a wavelength of 808 nm and was delivered through employment of a DMC laser system, specifically the Therapy XT model (Fig 5). The laser system exhibited a radiant power output of 100 mW, and the duration of irradiation for each distinct application site was precisely maintained at 40 s. The laser will be hovering close to the surface of the vaginal region. The detailed radiometric parameters can be observed in Table 1.

**Table 1:** Radiometric parameters.

Parameter	value
Peak wavelength:	808 nm
Operating Mode:	Continuous
Beam profile	Multimode
Polarization	random
Radiant power	100 mW
Radiant Energy:	4 J per point
Exposure duration:	40 seconds
Beam Area at target:	0.098 cm^2^
Irradiance:	1.016 W/cm^2^
Radiant exposure:	40.64 J/cm^2^
Application Mode:	Hovering close to the surface
Anatomical Location of Application:	8 points on the vulvar region
Treatment Sessions and Frequency:	Weekly, for 4 weeks
Total radiant energy per session:	32 J

**Fig 6:**
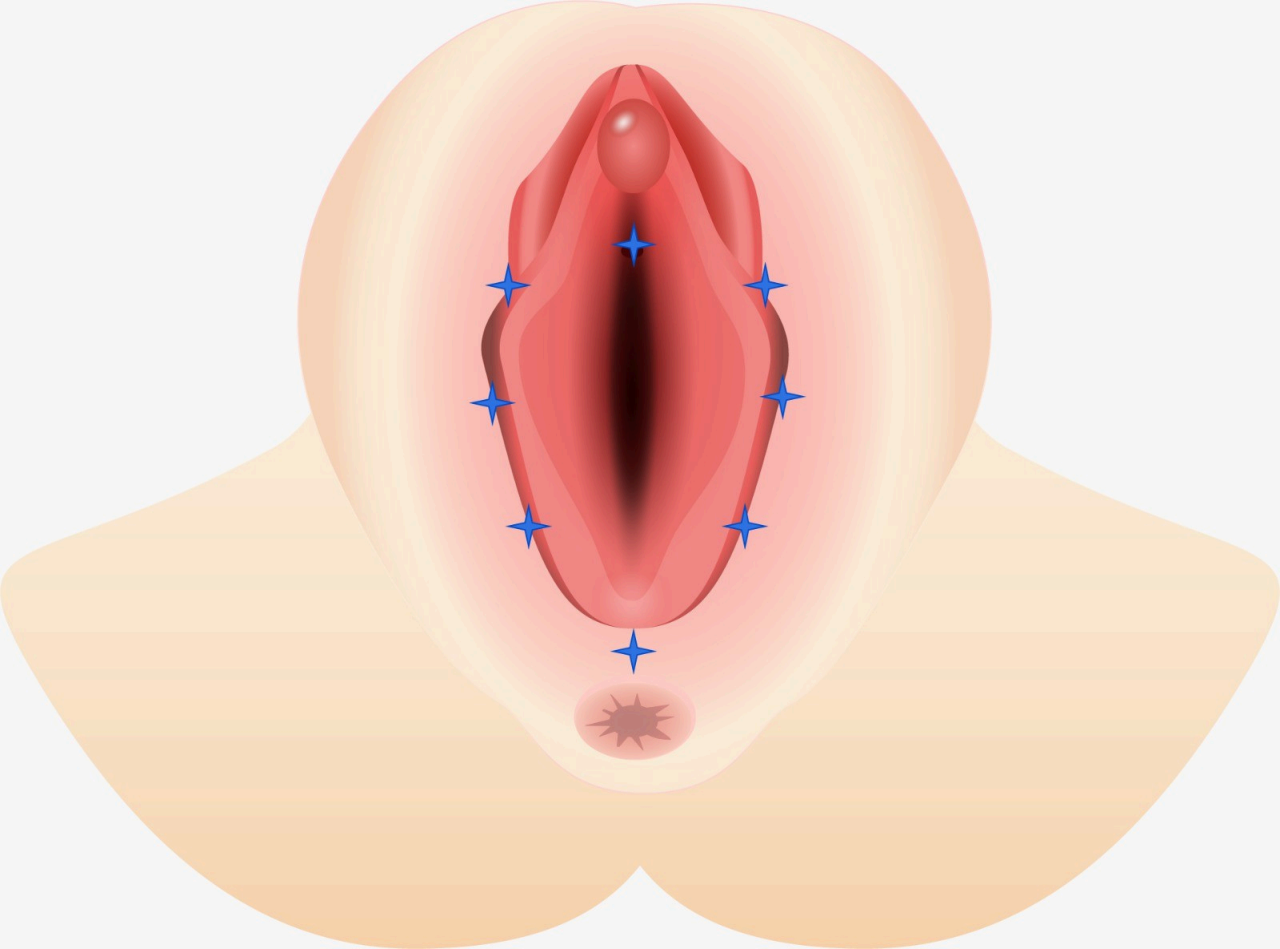
Anatomic sites irradiated.

Emanating from the established protocol, a total of eight distinct points distributed across the vulvar region were consistently irradiated in the context of each individual irradiation session, in strict adherence to the delineated methodology as described by Belotto *et al*. [39]. Fig 6 shows the anatomic sites irradiated.

The sham group underwent the same protocol, but the laser was turned off during the whole procedure.

At the end of the fourth session all subjects underwent a final pelvic pressure and urinary loss assessment.

## Discussion

The main objective of this study is to assess the effects of photobiomodulation in women exhibiting symptoms of genitourinary syndrome and urinary incontinence in the postmenopausal phase. Our hypothesis is that this protocol may ameliorate urinary incontinence and alleviate symptoms of postmenopausal genitourinary syndrome, offering a non-hormonal approach to treat this condition. This could potentially serve as a viable option for managing Genitourinary Syndrome of Menopause, contributing to the improvement of signs, symptoms, and quality of life during this phase of women’s lives. Furthermore, this approach may be considered for integration into the public healthcare system, as there are currently no available treatments for genitourinary syndrome in the public healthcare network.

## Study limitations

The main limitation of this study is the short evaluation period of 90 days, which may be insufficient to determine the long-term efficacy and durability of photobiomodulation therapy for treating GSM symptoms. Additionally, the reliance on self-reported data could potentially introduce bias due to subjective interpretations and reporting inaccuracies by participants.

## Supporting information

S1 Checklist SPIRIT 2013 checklist recommended items to address in a clinical trial protocol and related documents filled with the details of the project. (DOC)

S1 File (DOCX)
