# Supplementary material for: Photobiomodulation in post menopause genitourinary syndrome—Study protocol for a randomized, double-blind, controlled clinical protocol
Source: PLoS One. 2024 Dec 2;19(12):e0313324. doi: 10.1371/journal.pone.0313324 (PMC11611114; doi:10.1371/journal.pone.0313324)
Supplement: S1 Checklist — (DOC) [file pone.0313324.s001.doc]

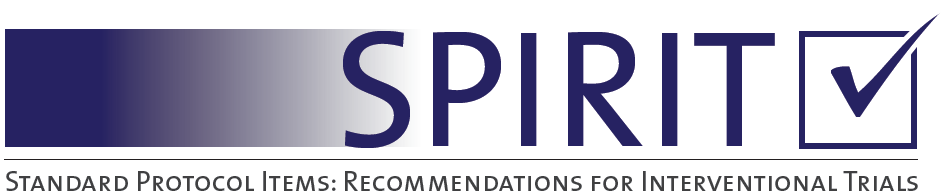


SPIRIT 2013 Checklist: Recommended items to address in a clinical trial protocol and related documents*

| Section/item | ItemNo | Description |
| --- | --- | --- |
| **Administrative information** | | |
| Title | 1 | Photobiomodulation in Post Menopause Genitourinary Syndrome - Study Protocol for a Randomized, Double-blind, Controlled Clinical Protocol. |
| Trial registration | 2 | This protocol is registered at ClinicalTrials.gov under the number NCT05557799. |
|  |  |  |
| Protocol version | 3 | It was first posted on 2022-09-28, https://clinicaltrials.gov/study/NCT05557799. |
| Funding | 4 | CAPES |
| Roles and responsibilities | 5a | Silvia Regina dos Santos Pereira1, Raquel Agnelli Mesquita-Ferrari1, Luciana Almeida-Lopes², Paulo Sérgio Bossini², Marcela Leticia Leal Gonçalves3, Alessandro Melo Deana1*  1. Post Graduation Program in Biophotonics Medicine, Universidade Nove de Julho, São Paulo, SP, Brazil.  2. Institute of Research and Education in the Health Area (NUPEN), São Carlos, SP, Brazil.  3. Post Graduation Program in Health and Environment, Universidade Metropolitana de Santos – UNIMES, Santos, SP, Brazil.  SRSP draft writing as well as principal research in charge of apply the protocol  RAMF, LAL, PSB and MLLG: protocol revision and proof-reading of the work  AMD: management of the work, revision and general advisor of the protocol |
|  | 5b | NUPEN institute. Endereço: R. Sebastião de Moraes, 800 - Jardim Alvorada, São Carlos - SP, 13562-030  Telefone: (+5516) 2107-3200 |
|  | 5c | This work was partially funded by NUPEN, but the sponsors had no management or decision role in this work. |
|  | 5d | NA |
| Introduction |  |  |
| Background and rationale | 6a | Genitourinary Syndrome of Menopause (GSM) defines a set of symptoms associated with an estrogen deficit involving alterations in organs genitourinary and that results in several urinary, genital, and sexual alterations. Brazilian women live about a third of their life after menopause, where hormonal changes occur along with clinical manifestations, characterized by vaginal and vulvar dryness, burning sensation, discomfort, vulvovaginal irritation, lack of lubrication, dyspareunia and urinary incontinence. Fractionated photothermolysis and radiofrequency systems, alone or in combination were tested to improve PGS. |
|  | 6b | In the given context, considering the state of the art as previously discussed and recognizing the limitations of current technology, there is a compelling necessity to innovative therapeutic approaches for the management of Genitourinary Syndrome of Menopause in women who suffer from urinary incontinence. Consequently, the aim of this study is to determine, through a randomized, placebo-controlled clinical trial, the influence of photobiomodulation on the clinical outcomes of participants exhibiting symptoms of genitourinary syndrome and urinary incontinence during the postmenopausal phase. |
| Objectives | 7 | To investigate the clinical response of postmenopausal participants with urinary incontinence after external vaginal photobiomodulation through a clinical trial. |
| Trial design | 8 | This study protocol was designed as a prospective, randomized, double-blind, controlled trial according to the 2023 SPIRIT (Standard Protocol Items: Recommendations for Interventional Trials) Statement and SPIRIT figure 2 and will be conducted at Nove de Julho University and the Municipal Health Department of Vargem Grande Paulista, SP in 2021–2023. |
| Methods: Participants, interventions, and outcomes | | |
| Study setting | 9 | Participant selection will be carried out among women attending routine appointments at healthcare facilities in the municipality of Vargem Grande Paulista. Additionally, we will also hold a meeting with elderly individuals at the Senior Citizens' Center in the municipality to identify potential participants |
| Eligibility criteria | 10 | **Inclusion:** women over 50 years old in the postmenopausal period (amenorrhea for at least 12 months without involved pathology), presenting one or more symptoms of GSM (such as dyspareunia, dryness, irritation, vaginal burning and/or discomfort, vaginal and vulvar atrophy, vaginal dryness, dysuria, polyuria, recurrent urinary infections), with complaints of stress and/or urgency urinary incontinence, presenting a normal cervical cytology (Pap smear) performed within the last year. **Exclusion:** woman on hormonal medication for menopausal symptoms in the last 6 months, participants who explicitly declined to participate, participants who had a history of bilateral oophorectomy, subjects with conditions such as recent Acute Myocardial Infarction (AMI), neoplasms, history of thrombosis, liver insufficiency, uncontrolled genital bleeding,genital condylomatosis, active genital herpes, subjects with surgeries of the lower genital tract that would preclude treatment. |
| Interventions | 11a | After the recruitment the subjects a gynecologic analysis and those who fulfill the inclusion criteria performed the first pelvic pressure and urinary loss measurements. After this they were randomly assigned to one of the study groups: sham or photobiomodulation.  The photobiomodulation cohort was subjected to the prescribed irradiation regimen in the subsequent manner. Each participating individual will undergo a standardized treatment protocol, involving a one irradiation session per week administered over a span of four consecutive weeks. During each individual session 4J of radiant energy per application site was imparted. This energy was emitted at a wavelength of 808 nm, and was delivered through employment of a DMC laser system, specifically the Therapy II model. The laser system exhibited a radiant power output of 100 mW, and the duration of irradiation for each distinct application site was precisely maintained at 40s. |
|  | 11b | If the participant interrupts a treatment session or chooses to discontinue their participation, the treatment will be suspended, and the participant's data will be removed from the study. However, they will continue to be monitored for safety assessment purposes. |
|  | 11c | The participants will be included in a phone message group through which they will be reminded of the follow-up appointments by the researchers. |
|  | 11d | No major harms are expected, but if possible intercurrent events occur, they will be monitored and recorded. Any additional assistance that participants may need will be provided. |
| Outcomes | 12 | The sample size was calculated using the formula described in the article "Sample size calculation," published in 2010 by Prashant Kadam and Supriya Bhalerao. Considering a type I error of 0.05 and a test power of 80%, the calculated sample size was 60 participants, with 30 in each group (A and B). The figure shows that, for medium (0.750) and large effects (1.000-1.250), a minimum of 30 patients per group is sufficient to control statistical variance, ensuring a test power greater than 0.80. Taking into account a 10% sample loss, a total of 66 patients will be recruited for the study. |
| Participant timeline | 13 | Time schedule of enrolment, interventions (including any run-ins and washouts), assessments, and visits for participants. A schematic diagram is highly recommended (see Figure)  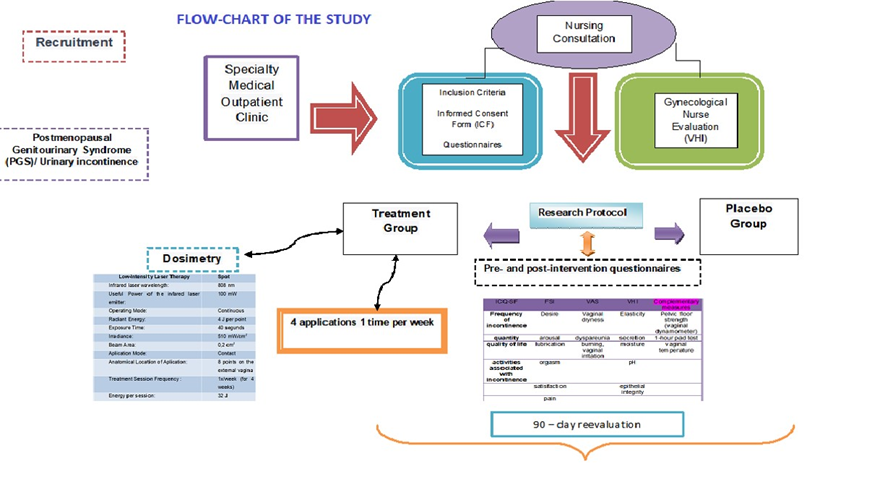  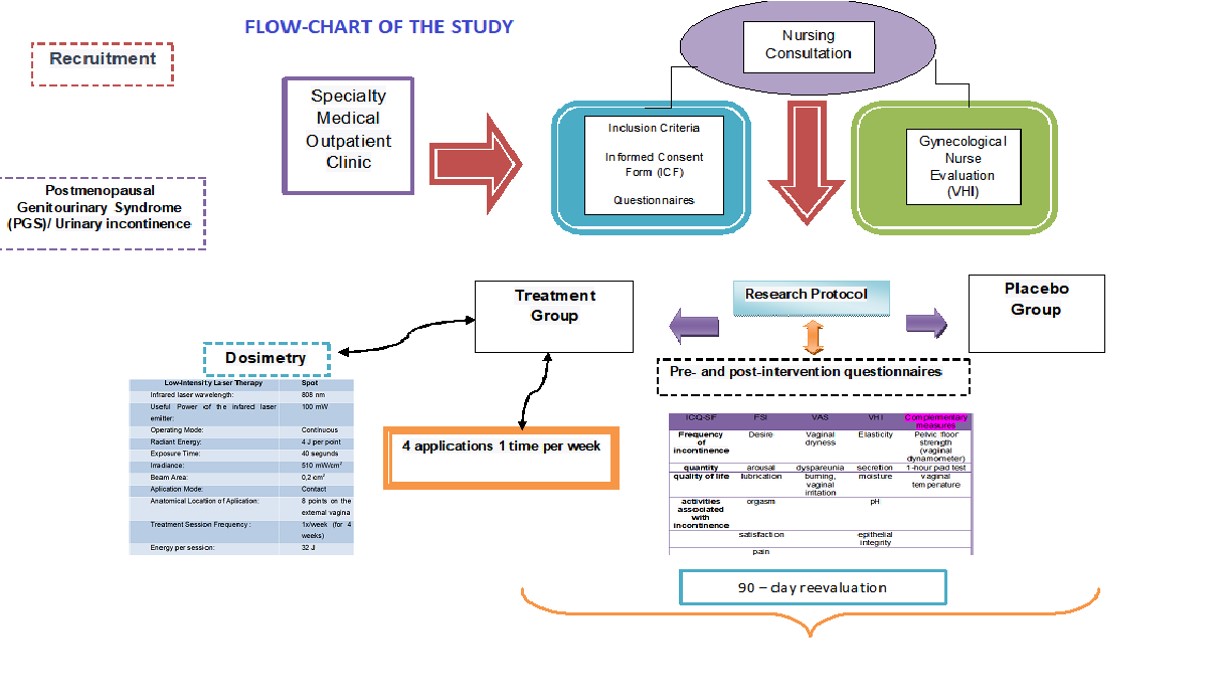  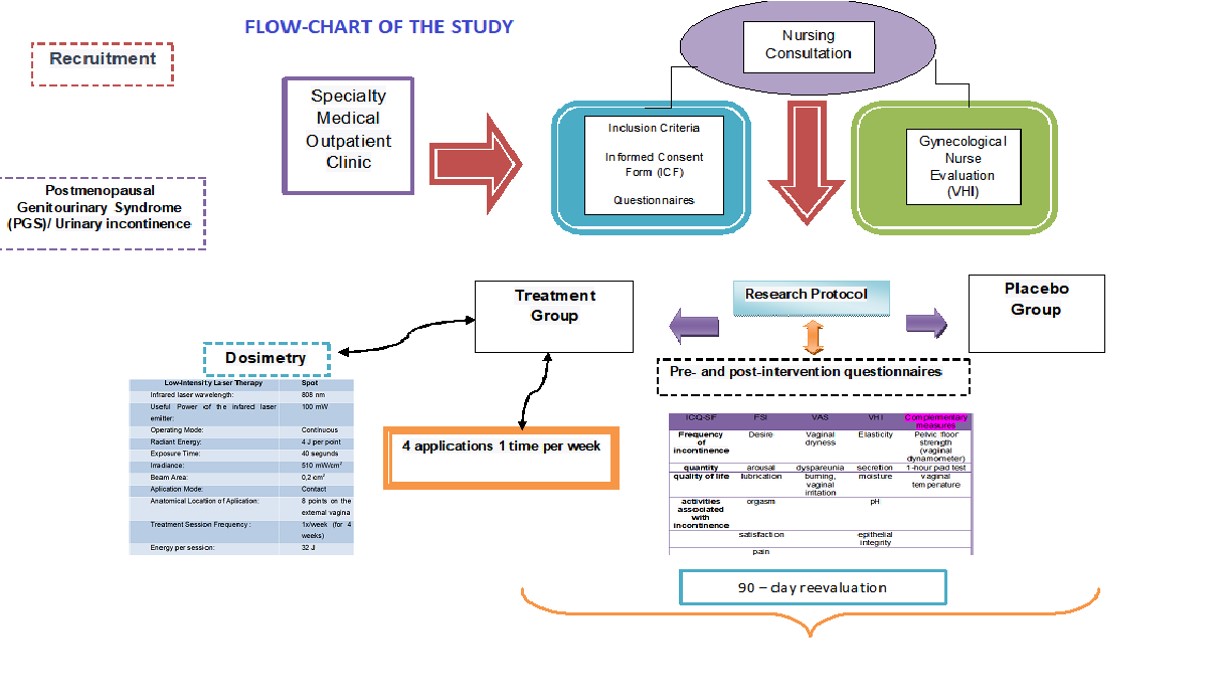 |
| Sample size | 14 | Since the literature lacks studies on the effects of the photobiomodulaton on GSM, the sample size was calculated using the formula described using the estimated effect size, as described by Kadam and Bhalerao. Considering a type I error of 0.05 and a test power of 80%, the calculated sample size was 60 participants, with 30 in each group (A and B). The figure shows that, for medium (0.750) and large effects (1.000-1.250), a minimum of 30 patients per group is sufficient to control statistical variance, ensuring a test power greater than 0.80. Considering a 10% of sample loss, a total of 66 patients will be recruited for the study. The figure 3 shows a plot of the test power as a function of the sample size and estimated effect size. |
| Recruitment | 15 | Recruitment will be conducted through the public health system of Vargem Grande Paulista, SP, Brazil. Additionally, recruitment efforts were carried out through the municipality's Senior Center, social media, and referrals from participants who had previously undergone the project. |
| **Methods: Assignment of interventions (for controlled trials)** | | |
| Allocation: |  |  |
| Sequence generation | 16a | The participants were randomly assigned to two groups, Group A (treatment) and Group B (placebo), in block-randomization fashion, with each block comprising four participants. After participant selection and assessment, sealed brown envelopes were randomly assigned to each subject containing the treatment allocation until the block was over, afterwards a new block of four allocation (two in each group) was made. |
| Allocation concealment mechanism | 16b | The participants Will be randomly allocated in blocks into two groups: Group A (treatment) and Group B (sham). Each block consisted of four participants. The patients Will be randomly allocated to two groups:   1. Study group (N = 30): was receive four consecutive applications, using laser diode DMC (808 nm), 4J per point, 100 mW of power, 510mW/cm², beam area of 0.2cm², 8 sites in the external vagina, for the 40s in each site, once per week for 4 weeks. 2. Sham group (N = 30): will be handled as treated, but with the laser turned off. |
| Implementation | 16c | All patients who provide consent for participation and meet the inclusion criteria will undergo randomization. The randomization process will be initiated by the designated staff member |
| Blinding (masking) | 17a | The evaluator of the VHI and the participants will be kept unaware of the allocation of participants to the groups. Exclusive awareness of whether the blue light exhibits a biological effect or serves as a placebo will be retained by the researcher. |
|  | 17b | If blinded, circumstances under which unblinding is permissible, and procedure for revealing a participant’s allocated intervention during the trial  NA |
| **Methods: Data collection, management, and analysis** | | |
| Data collection methods | 18a | The quality of life will be assessed using female sexual functioning index (FSFI-6), urinary incontinence questionnaire (ICIQ-SF), Quality of life will be analyzed using the female sexual functioning index (FSFI-6). The intensity of menopausal symptoms will be evaluated using a visual analogue scale (VAS), the vulvo vaginal atrophy will be measured by the Vaginal Health Index (VHI). Also, the vaginal temperature will be measured using a thermal camera, the pressure of the pelvic floor force (vaginal dynamometer) and a 1-hour Pad Test will be performed to quantify the urinary loss. |
|  | 18b | The participants will be included in a phone message group through which they will be reminded of the follow-up appointments by the researchers. |
| Data management | 19 | All data will be entered electronically, in Excel files, stored in computers and hard discs. The participants’ files will be accessible only to the authors of this study and only they will be able to edit the information, mantaining data accuracy and validation.. |
| Statistical methods | 20a | The data were tested for normality using the Shapiro-Wilk test, and those with parametric distribution were represented by their respective means and standard deviations. In cases where the assumption of parametric distribution was rejected, medians and quartiles were used. To assess the association between the studied groups and the independent variables, Student's t-test or Wilcoxon-Mann-Whitney test was used for parametric and non-parametric data, respectively. All tests were two-tailed, and the adopted significance level was α = 0.05. |
|  | 20b | Methods for any additional analyses (eg, subgroup and adjusted analyses)  NA |
|  | 20c | Definition of analysis population relating to protocol non-adherence (eg, as randomised analysis), and any statistical methods to handle missing data (eg, multiple imputation)  Missing data will be excluded form the trial. |
| **Methods: Monitoring** | | |
| Data monitoring | 21a | Seeing as this is not a study that involves significant safety concerns, risks or complexity, neither is it a multicenter study of long duration, no data monitoring committee was considered required. |
|  | 21b | The corresponding author make the final decision to terminate the trial, if necessary. This will only happen in case it becomes impossible to clinically attend the participants as, for example, in outbreaks of infectious diseases. The authors will have access to these interim results. |
| Harms | 22 | No significant harm is expected, but if any intercurrences occur, they will be monitored and recorded. Any additional assistance that participants may require will be provided. |
| Auditing | 23 | The monitoring will be carried out weekly by the corresponding author responsible for the research. |
| Ethics and dissemination | | |
| Research ethics approval | 24 | This study is a pilot placebo-controlled, randomized, and double-blind clinical trial, approved by the Research Ethics Committee of Nove de Julho University (Uninove) number 5.628.333 and approved by the Municipal Health Department of Vargem Grande Paulista, SP. |
| Protocol amendments | 25 | Plans for communicating important protocol modifications (eg, changes to eligibility criteria, outcomes, analyses) to relevant parties (eg, investigators, REC/IRBs, trial participants, trial registries, journals, regulators) |
| Consent or assent | 26a | Any modifications to the protocol which may impact on the conduct of the study, potential benefit of the patient or may affect patient safety, including changes of study objectives, study design, patient population, sample sizes, study procedures, or significant administrative aspects will require a formal amendment to the protocol. Such amendment will be agreed upon by the Ethics in human research of the universidade Nove de Julho and the Department of the municipality of Vargem Grande Paulista – SP. |
|  | 26b | no applicable |
| Confidentiality | 27 | The data sets generated and analyzed during the study will be available from the corresponding author at reasonable request. Once the data is entered electronically, participants identification details will no longer be attached to their data, they will be reported only by codes. After the analysis of the data, volunteers will be invited to a meeting and the results will be shared, in case they wish to attend it. The authors also intend to publish the results. |
| Declaration of interests | 28 | The authors declare that they have no competing interests. |
| Access to data | 29 | The data sets generated and analyzed during the study will be available from the corresponding author at reasonable request. After the analysis of the data, volunteers will be invited to a meeting and the results will be shared, in case they wish to attend it. The authors also intend to publish the results. |
| Ancillary and post-trial care | 30 | If this study demonstrates the efficacy of photobiomodulation in the external vaginal area and leads to a reduction and/or elimination of urinary incontinence in postmenopausal women, it is of utmost importance to ensure timely access to the treatment. As part of the preparations for this study, discussions have been initiated with the Health Department of Vargem Grande Paulista, the Nove de Julho University, and DMC Laser Equipments to facilitate this access. The discussions encompass issues such as licensing agreements and preferential pricing for the study communities, as well as for other regions with limited resources. |
| Dissemination policy | 31a | After the analysis of the data, volunteers will be invited to a meeting and the results will be shared, in case they wish to attend it. The authors also intend to publish the results. |
|  | 31b | The final datasets will be made available by the corresponding author upon reasonable request. The authors intend to disseminate the results through articles and conference presentations. |
|  | 31c | Plans, if any, for granting public access to the full protocol, participant-level dataset, and statistical code  NA |
| Appendices |  |  |
| Informed consent materials | 32 | Model consent form and other related documentation given to participants and authorised surrogates  Attached to the submission |
| Biological specimens | 33 | no applicable |

*It is strongly recommended that this checklist be read in conjunction with the SPIRIT 2013 Explanation & Elaboration for important clarification on the items. Amendments to the protocol should be tracked and dated. The SPIRIT checklist is copyrighted by the SPIRIT Group under the Creative Commons “[Attribution-NonCommercial-NoDerivs 3.0 Unported](http://www.creativecommons.org/licenses/by-nc-nd/3.0/)” license.
