# Supplementary material for: Photobiomodulation in post menopause genitourinary syndrome—Study protocol for a randomized, double-blind, controlled clinical protocol
Source: PLoS One. 2024 Dec 2;19(12):e0313324. doi: 10.1371/journal.pone.0313324 (PMC11611114; doi:10.1371/journal.pone.0313324)
Supplement: S1 File — (DOCX) [file pone.0313324.s002.docx]

universidade nove de julho

PROGRAMA DE PÓS-GRADUAÇÃO EM BIOFOTÔNICA APLICADA ÀS

CIÊNCIAS DA SAÚDE

Silvia Regina dos Santos Pereira

**Fotobiomodulação na Síndrome Geniturinária PÓS menopausa**

são paulo

2022

universidade nove de julho

PROGRAMA DE PÓS-GRADUAÇÃO EM BIOFOTÔNICA APLICADA ÀS

CIÊNCIAS DA SAÚDE

Silvia Regina dos Santos Pereira

**Fotobiomodulação na Síndrome Geniturinária PÓS menopausa**

Projeto de Pesquisa apresentado ao Programa de Pós-Graduação em Biofotônica aplicada às Ciências da Saúde da Universidade Nove de Julho - UNINOVE, como requisito parcial para a obtenção do grau de Doutor em Biofotônica.

são paulo

2022

# RESUMO

A Síndrome Genitourinária da menopausa é causada pelo hipoestrogenismo fisiológico do climatério e resulta em várias alterações urinárias, genitais e sexuais. As mulheres brasileiras vivem cerca de um terço da vida após a menopausa, onde ocorrem alterações hormonais juntamente com manifestações clínicas, caracterizadas por secura vaginal e vulvar, queimação, desconforto, irritação vulvovaginal, falta de lubrificação, dispareunia, disúria, polaciúria e infecções urinárias recorrentes. Fototermólise fracionada e sistemas de radiofrequência, sozinhos ou em combinação, foram testados para melhorar a SGU na menopausa.O objetivo deste projeto é avaliar a resposta clínica de pacientes com sintomas da síndrome pós menopausa geniturinária após a aplicação de fotobiomodulação na vagina e sua introdução.A População será de 60 mulheres, com 50 anos ou mais de idade, com queixas de SGU e incontinência urinária pós-menopausa, que serão randomizadas de acordo com dois grupos (controle e tratamento placebo). Neste protocolo de estudo randomizado, duplo-cego e controlado por placebo. O grupo tratamento (n = 30) receberá quatro aplicações consecutivas, utilizando diodo laser DMC (808 nm), 4J por ponto, 100mW de potência, 510mW / cm², área de feixe de 0,2cm², 8 sítios na vagina externa, para o 40s em cada local, uma vez por semana durante 4 semanas. O Grupo Placebo (n = 30) será tratado como o grupo tratado, mas com o laser desligado. A qualidade de vida será analisada por meio do índice de funcionamento sexual feminino (FSFI-6), do questionário de incontinência urinária (ICIQ-SF), a intensidade dos sintomas da menopausa será avaliado através de uma escala visual analógica (VAS), a atrofia vulvo gavinal será mensurada pelo Índice de Saúde Vaginal (VHI) e comparada entre os grupos. Além disso, a temperatura vaginal será medida por meio de um termômetro digital, a pressão de força do assoalho pélvico (dinamômetro vaginal) e um Pad Test de 1 hora será realizado para quantificar a perda urinária. Os dados serão testados quanto à sua normalidade utilizando o teste de Shapiro Wilks e, caso apresentem distribuição paramétrica serão representados por meio de suas respectivas médias e desvios padrão. Com este procedimento, pretendemos obter uma melhor qualidade de vida geral e diminuição dos sintomas em mulheres com SGU.

**Descritores**: Menopausa, Terapia com luz de baixa intensidade, Fotobiomodulação.

ABSTRACT

**INTRODUCTION:** Postmenopausal Genitourinary Syndrome (PGS) defines a set of and signs associated with an estrogen deficit involving alterations in organs genitourinary and that results in several urinary, genital, and sexual alterations. Brazilian women live about a third of their life after menopause, where hormonal changes occur along with clinical manifestations, characterized by vaginal and vulvar dryness, burning, discomfort, vulvovaginal irritation, lack of lubrication, dyspareunia, dysuria, pollakiuria, and recurrent urinary infections. Fractionated photothermolysis and radiofrequency systems, alone or in combination were tested to improve PGS. **OBJECTIVE:** The goal of this project is to evaluate the clinical response of patients with symptoms of genitourinary menopause syndrome after the application of photobiomodulation in the vagina and its introit. **METHOD**: In this randomized, double-blind, placebo-controlled study protocol. Women over 50 years of age who are in the postmenopausal period (amenorrhea for at least 12 months, with no pathology involved) with one or more symptoms of PGS.Participants included in the study will be randomly divided into two groups: group A, which will receive photobiomodulation with a vaginal diode laser and its introit and group B (placebo) with the laser device turned off. Both treatments will be maintained for 4 consecutive weeks, as shown in Figure 1.The treatment group (n=30) will receive four consecutive applications, using laser diode DMC (808 nm), 4J per point, 100mW of power, 510mW/cm², beam area of 0.2cm², 8 sites in the external vagina, for the 40s in each site, once per week for 4 weeks. The Placebo Group (n=30) will be handled as treated, but with the laser turned off. The life quality will be analyzed by using female sexual functioning index (FSFI-6), urinary incontinence questionnaire (ICIQ-SF), Quality of life will be analyzed using the female sexual functioning index (FSFI-6), the urinary incontinence questionnaire (ICIQ-SF), the intensity of menopausal symptoms will be evaluated using a visual analogue scale (VAS), the vulvo vaginal atrophy will be measured by the Vaginal Health Index (VHI) and compared between groups. Also, the vaginal temperature will be measured using a thermal camera, the pressure of the pelvic floor force (vaginal dynamometer) and a 1-hour Pad Test performed to quantify the urinary loss. The data will be tested for normality using the Shapiro Wilks test and, if they present a parametric distribution, they will be represented by means of their respective means and standard deviations. With this procedure, we intend to obtain an overall better life quality and diminished symptoms in women with PGS.

**Keywords: Menopause, Low-Level Light Therapy, Photobiomodulation.**

SUMÁRIO

[RESUMO 3](#_Toc95937822)

[ABSTRACT 4](#_Toc95937823)

[ÍNDICE DE FIGURAS 7](#_Toc95937824)

[1.CONTEXTUALIZAÇÃO DA TEMÁTICA 8](#_Toc95937825)

[1.2. REVISÃO LITERÁRIA SOBRE O APARELHO GENITAL FEMININO: 10](#_Toc95937826)

[1.3 Climatério e Menopausa: 12](#_Toc95937827)

[1.4. Diagnóstico da Menopausa: 19](#_Toc95937828)

[1.5. Tratamentos da Menopausa: 21](#_Toc95937829)

[1.5.1. Terapia Hormonal: 21](#_Toc95937830)

[1.5.2. Terapias Não Hormonais: 24](#_Toc95937831)

[1.6. Laser: 25](#_Toc95937832)

[1.6.1. Plataformas de Laser de Alta Potência: 27](#_Toc95937833)

[1.6.2. Efeitos do Laser sobre o Epitélio Vaginal 29](#_Toc95937834)

[1.6.3. Contra Indicações Laser Vaginal 30](#_Toc95937835)

[1.6.4. Efeitos Adversos e Complicações do uso do Laser Vaginal 31](#_Toc95937836)

[2. JUSTIFICATIVA: 32](#_Toc95937837)

[3. HIPÓTESES: 33](#_Toc95937838)

[4. OBJETIVOS: 34](#_Toc95937839)

[4.1. GERAL: 34](#_Toc95937840)

[4.2. ESPECÍFICOS: 34](#_Toc95937841)

[5. METODOLOGIA: 35](#_Toc95937842)

[5.1 Tipo de Estudo: 35](#_Toc95937843)

[5.2 Recrutamento: 35](#_Toc95937844)

[5.3. Critérios de inclusão e exclusão: 35](#_Toc95937845)

[5.4. Métodos de Avaliação da SGU na Menopausa: 36](#_Toc95937846)

[5.5. Benefícios Esperados: 42](#_Toc95937847)

[5.6. Riscos: 43](#_Toc95937848)

[5.7. Cálculo Amostral: 43](#_Toc95937849)

[5.8. Organização e Tratamento Estatístico dos Dados 43](#_Toc95937850)

[5.9. Randomização: 44](#_Toc95937851)

[5.9.1. Fluxograma da Pesquisa 44](#_Toc95937852)

[5.9. 2. Determinação dos grupos e intervenções: 44](#_Toc95937853)

[5.9.3. Etapas do Procedimento de Intervenção: 46](#_Toc95937854)

[5.9.4. ANÁLISE DOS DADOS: 47](#_Toc95937855)

[6. CRONOGRAMA: 48](#_Toc95937856)

[7. CONSIDERAÇÕES FINAIS 49](#_Toc95937857)

[REFERÊNCIAS: 50](#_Toc95937858)

# ÍNDICE DE FIGURAS

[Figura 1 Sistema Genital Feminino. 11](#_Toc94530681)

[Figura 2 - Comparação de uma vaginal saudável e atrofiada. . 17](#_Toc94530682)

[Figura 3 - Estágios da Idade Reprodutiva 21](#_Toc94530683)

[Figura 4 - Principais Cromóforos dos tecidos biológicos 27](#_Toc94530684)

[Figura 5 - Principais Plataformas de Laser. 29](#_Toc94530685)

[Figura 8 - Histologia da Mucosa Vaginal 1 mês após LCO2F 30](#_Toc94530688)

[Figura 9 - Mucosa Vaginal após 1 hora da aplicação do laser 30](#_Toc94530689)

[Figura 10 - Questionário ICQ- SF 38](#_Toc94530690)

[Figura 11 - Fita Indicadora de pH Vaginal 39](#_Toc94530691)

[Figura 12 - Dinamômetro Vaginal 41](#_Toc94530692)

[Figura 13 - Termômetro Digital Infravermelho 42](#_Toc94530693)

[Figura 14 – Análise do poder do teste em função do effect size e do tamanho amostral 43](#_Toc94530694)

[Figura 15 - Pontos de aplicação do laser vaginal e Locais de verificação da Temperatura na vulva. 45](#_Toc94530695)

[Figura 16 - Fluxograma de Pesquisa 47](#_Toc94530696)

#

# 1.CONTEXTUALIZATION OF THE THEME:

The Genitourinary Syndrome of Menopause (GSM) is a term coined in May 2013 by the ISSWSH (International Society for the Study of Women's Sexual Health) and NAMS (North American Menopause Society). It refers to a collection of symptoms and signs linked to an estrogen deficiency, involving changes in the genitourinary organs that lead to various alterations in urinary, genital, and sexual functions.^[[1]](#endnote-1)^.

In Brazil, the life expectancy for women is 76 years, and they comprise over 98 million people, representing the majority of users within the Unified Health System, accounting for approximately 50.77%. About 26,926,448 women are aged 40 and above. The Brazilian woman lives about one-third of her life post-menopause, a period marked by hormonal changes and various clinical manifestations.^[[2]](#endnote-2),^^[[3]](#endnote-3)^.

The syndrome or its characteristics manifest in approximately 15% of women in pre-menopause and 40-54% of women in post-menopause. Considering that women have a higher life expectancy than men, it is projected that by 2030, the female population over 65 years old will be around 17%. The consequences of the reduction in endogenous estrogen levels in menopausal women should be of significant interest to public health^[[4]](#endnote-4)^,^[[5]](#endnote-5)^,^[[6]](#endnote-6)^.

In the United States, around 50% of women in this phase report symptoms related to vaginal atrophy. It is estimated that Genitourinary Syndrome of Menopause (GSM) occurs in 8% to 22% of women in pre-menopause and 40% to 57% of women in post-menopause. However, only 1 in 5 women experiencing these symptoms have sought medical advice regarding GSM-related issues.^[[7]](#endnote-7)^.

International literature indicates that only 59% of these women seek medical help. In Europe, only 4% of women can report vulvovaginal symptoms, and approximately 25% of women with Genitourinary Syndrome of Menopause (GSM) consult a doctor. Additionally, 54% of women discuss their sexual health with healthcare professionals when prompted, while 33% of women do not address this issue during medical appointments.^5^.

The climacteric begins a few years before menopause and extends for a few years afterward (post-menopause). This transition does not represent diseases but rather states characterized by estrogen deficiency and aging phenomena. During this period, changes in the skin and genital trophism occur due to a combination of intrinsic and extrinsic factors. Genitourinary syndrome is a common complaint during this phase and has a significant impact on the quality of life of this population.^[[8]](#endnote-8)^.

A study in Venezuela involving 2,339 women found that 83% exhibited symptoms corresponding to the climacteric syndrome, with 49.3% experiencing severe symptoms, 31.0% moderate symptoms, and 19.7% mild symptoms. In Spain, it is estimated that over 50% of women in the climacteric experience a decrease in their quality of life due to the changes that occur during the peri-menopausal period.^[[9]](#endnote-9)^.

In Brazil, studies support these statistics, indicating that only 59% of women experiencing symptoms of urogenital atrophy seek medical help. Complaints of vaginal dryness are present in 34.9%, and respondents noted that being in the climacteric negatively impacted their sexuality. In the United States, 59% reported sexual dissatisfaction^[[10]](#endnote-10),^^[[11]](#endnote-11),^^[[12]](#endnote-12),^^[[13]](#endnote-13)^.

Hot flashes are symptoms that often disappear even without treatment, but issues associated with genitourinary syndrome increase with age and do not regress spontaneously. This leads women to experience prolonged and silent suffering. These chronic medical conditions impact mood, self-esteem, and hinder sexuality, making the emergence of sexual dysfunctions more likely.^[[14]](#endnote-14)^.

Regarding signs and symptoms, studies have identified a high frequency of complaints: dyspareunia (44%), irritation (37%), vaginal dryness (55%), recurrent urinary tract infections (5% to 17%), and urge incontinence in women over 60 years old (15% to 35%). For atrophic vaginitis, the prevalence was 4%, increasing to 25% in the first year after menopause and 47% after three years. Urinary complaints, urgency, and recurrent infections affect 5% to 17% of postmenopausal women, while dyspareunia has been observed to reduce the frequency of sexual relations, with 72% having intercourse once a month and 34% weekly.^[[15]](#endnote-15),^^[[16]](#endnote-16),1^.

Parish et al. published in their research that 80% of women learn to cope with the syndrome as the years pass. Among them, 30% reported that pain requires a pause or discontinuation of sexual activity. Additionally, 56% stated that they were sexually less active as a result of the pain, while 6% mentioned that pain prevented any sexual activity. Dyspareunia was present in 64% of postmenopausal women, 64% experienced a loss of libido, and 58% avoided any sexual intimacy.^[[17]](#endnote-17)^. Another perspective described in the literature is that women silently endure dyspareunia because they believe that vaginal atrophy is a natural part of aging, and they should learn to live with this disorder. The studies have found that they often do not report their complaints, and consequently, they do not receive treatment. This condition is underdiagnosed and undertreated due to a lack of communication between women and healthcare professionals. ^4,14^.

A genitourinary syndrome can vary in severity, ranging from discomfort to vaginal stenosis, making treatment essential for many women. The primary goal of treatment is to alleviate these bothersome symptoms. Therapeutic options for addressing this syndrome depend on their effectiveness, the severity of symptoms, and the woman's preferences. Regardless of the chosen treatment, regular sexual activity is considered a protective factor against vaginal atrophy. It is believed that sexual activity enhances blood flow to the pelvic organs, maintaining the elasticity of the mucosa and vaginal walls.^[[18]](#endnote-18)^.

There are various therapeutic options available for Atrophic Vaginitis, including hormonal and non-hormonal products. Among the non-hormonal devices are lubricants and moisturizers, which can be useful in cases of mild to moderate vaginal dryness. However, while they provide temporary relief of symptoms, these products may interfere with the spontaneity of sexual relations and have practical challenges in terms of application, resulting in low long-term adherence.

Regarding hormonal treatments, up to this point, the topical application of estrogens has been considered the "gold standard" treatment for moderate to severe cases or cases unresponsive to non-hormonal treatments.^17^.

The literature indicates that topical hormone therapy has some limitations. The beneficial effect is only evident during the treatment period, with high recurrence rates upon discontinuation. Additionally, it only acts on the surface of the vaginal epithelium, lacking effects on the renewal of the extracellular matrix (ECM) or vascularization. Another important factor to note is that many women may discontinue the treatment due to concerns about its long-term consequences, given the risk of developing hormone-dependent neoplasms.^[[19]](#endnote-19),^^[[20]](#endnote-20)^.

The medical adherence rate to this line of therapy is quite variable (52-74%), mainly due to safety concerns, inconvenience, and inadequate relief of symptoms from available treatments. The AGATA study demonstrated that only 2.9% of 274 women diagnosed with Genitourinary Syndrome of Menopause (GSM) were undergoing treatment at the beginning of the research. One of the reasons for not seeking treatment is dissatisfaction with the results.^[[21]](#endnote-21)^.

The literature supports the efficacy of laser therapy, a new, practical, and safe technology for the treatment of Genitourinary Syndrome of Menopause (GSM). Laser therapy is painless, well-tolerated, and can be applied on an outpatient basis. When compared to the topical application of estrogens, considered the "gold standard" therapy, laser therapy appears to be an alternative with longer-lasting and equally or more effective effects.

The potential of laser therapy becomes highly relevant for women with Atrophic Vaginitis (AV) and a history of hormone-dependent neoplasms, where estrogen therapy may be contraindicated.^[[22]](#endnote-22)^. However, several studies point out certain limitations in research on the use of lasers for Genitourinary Syndrome of Menopause (GSM), such as a scarcity of randomized controlled trials, larger sample sizes, the use of placebo laser devices compared to groups undergoing hormone therapy, longer follow-up periods, selection biases due to participation by women dissatisfied with hormonal treatments, and potential conflicts of interest as laser companies sponsor the research.^15, 18, 19, 20, 21, 22^.

Studies indicate that the use of CO2 lasers, fractional lasers, Erbium: YAG lasers, and radiofrequency systems, either individually or in combined therapy, improves Genitourinary Syndrome of Menopause (GSM). These treatments induce morphological changes in vaginal tissues and increased vaginal blood circulation, leading to improvements in complaints of vaginal dryness, dyspareunia, and stress urinary incontinence.^[[23]](#endnote-23)^. However, this method has a high cost and is not available in the Unified Health System (SUS), which has an impact on public health. ^[[24]](#endnote-24)^.

## 1.2. LITERATURE REVIEW ON THE FEMALE GENITAL SYSTEM:

The female genital system is divided into external and internal genitalia (Figure 1). The external genitalia, or vulva, is comprised of the vulvoperineal region.


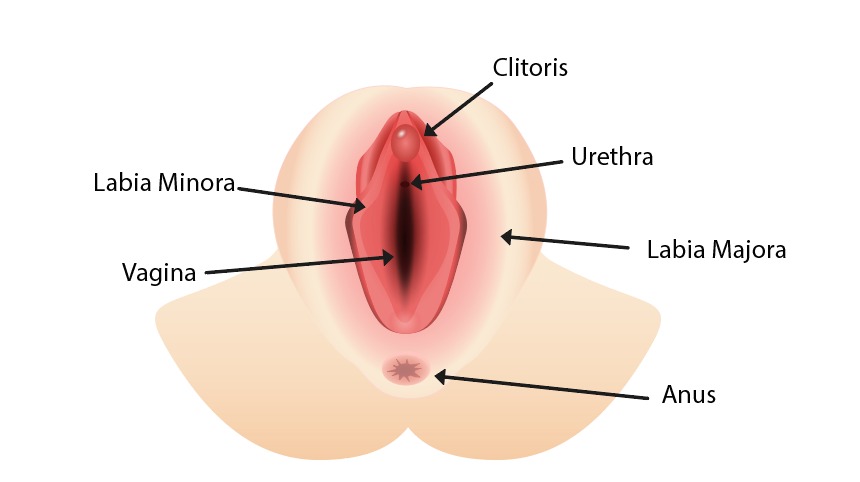


Figura 1 Female Genital System. Source: own.

The vulva represents the entrance to the vagina, covering and protecting the urethral meatus. It is covered by hair follicles, sebaceous glands, and sweat glands. Internally, the skin undergoes modifications, becoming moist and hairless.^[[25]](#endnote-25)^.

The vagina is the canal that connects the internal and external female genital organs. Its function is reproductive and sexual. It is composed of a fibrous tunic that surrounds a muscular tunic and is internally lined with a mucosal tunic. The muscular portion consists of layers of smooth, longitudinal, and circular muscle fibers. The submucosal region has a dense network of veins and lymphatic vessels. The entire mucosal surface is folded with transverse and oblique vaginal rugae, projecting internally. The vaginal wall extends from the uterus to the external vestibule, measuring 7 to 14 cm in length and 4.7 to 6.3 cm in width. The blind-ended circular structure formed around the uterus is called the vaginal fornix and is divided into anterior, posterior, and lateral fornices. The vaginal introitus measures from 2.4 to 6.5 cm.^[[26]](#endnote-26)^.

The anterior part of the vagina is in contact with the urethra, bladder, and more superiorly, the ureters. The posterior fornix of the vaginal fornix is covered by the peritoneum of the recto-uterine pouch. Below, the vagina is in contact with the rectum, separated only by a thin layer of areolar connective tissue. As it approaches the vestibule, the rectum moves away, and this space is occupied by muscle fibers, adipose tissue, and connective tissue, forming the perineal body. The vagina is supported by the cardinal or transverse ligaments in the upper portion, by the levator ani or puborectalis muscle in the lower third, and by the bulbospongiosus muscles and bodies at the introitus.^26^.

A vascular supply to the vagina is anteriorly provided by the vaginal branch of the uterine artery and posteriorly by branches of the middle and inferior rectal arteries. The veins follow the course of the arteries. Lymphatic vessels in the mucosa are numerous and anastomose with deeper muscular vessels. The upper lymphatic vessels accompany the uterine artery and terminate in the external iliac lymph nodes. The middle portion's lymphatic vessels, draining the majority of the vagina, follow the vaginal arteries to the hypogastric canal. Lymph nodes of the rectovaginal septum and lower lymphatic vessels either accompany those from the middle portion or drain into inguinal lymph nodes.^26^.

The vaginal mucosa has a thickness of 150 to 200µm and is covered by non-keratinized stratified squamous epithelium, rich in glycogen. Glycogen transforms into glucose, producing lactic acid through the action of the vaginal flora, maintaining the pH between 3.8 and 4.2. The vagina lacks glands. The mucus formed in the lumen originates from the glands of the uterine cervix, vaginal desquamation, and transudate from sexual stimulation. The vaginal wall is composed of three layers: adventitia, muscular, and mucosa.^[[27]](#endnote-27)^.

The vaginal mucosa consists of non-keratinized stratified squamous epithelium with a large number of neutrophils, lymphocytes, and Langerhans cells. It has a visually rough appearance. The lamina propria of the vaginal mucosa is composed of loose connective tissue rich in elastic fibers. Although well-vascularized, it has few sensory nerve endings. This mucosa is responsible for the nourishment, lining, and architecture of the vaginal wall.^[[28]](#endnote-28)^.

The elastic fibers in this wall, composed of collagen and elastin, participate in controlling the biomechanical properties of vaginal tissue. The main subtypes of collagen present in the vagina are type I (large and strong fibers), type III (smaller fibers with less tensile strength and more elastic), and type V (small fibers with low tensile strength located in the core of the fibril). Polymerization between them forms mixed fibers, which determine the mechanical strength of the vaginal wall.^[[29]](#endnote-29)^.

## 1.3 Climacteric and Menopause:

A woman undergoes different hormonal cycles throughout her life, beginning with puberty (menarche) and extending until menopause when cyclicality ceases. The release of female hormones by the ovaries, under pituitary stimulation, defines the various periods of the female hormonal cycle. Estrogen and progesterone are crucial hormones in the female biological cycle and influence secondary sexual characteristics, ovum release, maintenance of pregnancy, and female behavior.^[[30]](#endnote-30)^.

We are born with seven million ovarian follicles, and over 99% of them undergo atrophy during a woman's life. Menopause is a consequence of this process, marked by a decrease in ovarian production of estrogen and progesterone, accompanied by amenorrhea, which occurs around the age of 51. During this process, many women ovulate irregularly due to a decline in estrogen levels, leading to insufficient corpus luteum and a deficit of progesterone or follicular resistance to ovulatory stimulation. Menopause is defined as the cessation of menstruation (for a period of twelve months of amenorrhea), either as a normal part of aging or as a result of surgical removal of both ovaries, marking a significant milestone in the climacteric. The term "menopause" signifies the end of menstruation.^[[31]](#endnote-31),^^[[32]](#endnote-32)^.

The climacteric is the phase of a woman's life characterized by the transition from the reproductive to the non-reproductive period. It can be divided into three parts: premenopause (the period leading up to the end of menstruation in a woman's life), perimenopause (a period of 3 to 5 years preceding the last menstruation and extending one year after), and postmenopause (the interval between the last menstrual bleeding and old age).^[[33]](#endnote-33)^.

Menopause is not a disease, but it can trigger signs and symptoms that require control and evaluation to ensure the quality of life during the female aging process. ^[[34]](#endnote-34)^. This process occurs due to a reduction in the activity of the ovaries, which cease to release eggs monthly. At the same time, estrogen is secreted in smaller quantities. This stage is called hypoestrogenism. If this occurs suddenly, women may experience more prominent symptoms than if the onset is slow and progressive.^[[35]](#endnote-35)^.

A hypoestrogenemia causes a systemic repercussion, leading to signs and symptoms that characterize menopause, which can be didactically divided into early and late manifestations. Estrogen, a chemical mediator produced by the ovaries from cholesterol, acts on both reproductive and non-reproductive organs during the reproductive years. Moreover, it has specific receptors in different cells, triggering cellular responses in various tissues, maintaining the organic and emotional functions of women.^30^. Estrogen favors female biopower, stimulating the production of collagen and elastin, the action of fibroblasts contributing to skin hydration and elasticity, making the skin smooth. It influences voice, mood, appetite reduction, improvement of smell, increases physical disposition, provides silkier hair, acts on body anatomy, making women perceive themselves as more attractive and beautiful, thereby arousing interest in sex.^[[36]](#endnote-36)^.

- **Vasomotor manifestations:** hot flashes, increased heart rate, sweating, palpitations, distress, and anxiety.;

This physiopathological mechanism appears to be related to an alteration at the level of brain neurotransmitters, caused by a decrease in estrogen. There is an increased release of Gn-Rh (which stimulates the pituitary production of LH and FSH) and a disturbance in thermal balance. They manifest as a typically intense hot flash that predominantly affects the upper half of the body, followed by cold sweats within a few minutes. They are accompanied by an increase in heart rate and peripheral blood flow. Sometimes, they are associated with dizziness. They are not controllable or predictable by the woman.^35^.

Vasomotor symptoms are associated with increased blood flow and heart rate and are perceived in 80% of menopausal women due to changes in neurotransmitter levels in the brain.^[[37]](#endnote-37)^.

Episodes of hot flashes can last from 30 seconds to 5 minutes and may be followed by chills. Hot flashes can occur at night in the form of night sweats. The mechanism behind this is unknown, but it is believed to result from changes in the thermoregulatory center located in the hypothalamus. The range of basic body temperatures that is comfortable for women decreases; therefore, a slight change in body temperature can trigger the release of heat as a hot flash.^[[38]](#endnote-38)^.

A Sociedade Brasileira de Climatério (SOBRAC) describes that during hot flashes, the skin temperature can increase between 1 and 7°C. A sudden wave of heat spreads throughout the body, particularly in the upper body and face. The following phenomenon involves significant sweating, causing the woman to feel cold shortly after a heat wave. It can also occur when the woman sleeps, leading to night sweats. Night sweats are the second most frequent symptom of perimenopause, experienced by approximately 75% of women, and are considered the hallmark of perimenopause. Their frequency does not follow a specific pattern, occurring daily, weekly, or even monthly. The duration of hot flashes is typically 3 to 5 years, and in surgical menopause, they may be more severe.^[[39]](#endnote-39)^

**• Menstrual Dysfunctions in Climacteric:**

Menstrual cycle dysfunctions begin in the transition from climacteric to menopause due to a decrease in estrogen levels, resulting in clinical manifestations that vary in each woman. Some manifestations described in the literature include: hypermenorrhea - menstrual bleeding that lasts more than five days; hypomenorrhea - when menstrual bleeding lasts less than two days; menorrhagia - heavy menstrual bleeding; oligomenorrhea - when there is a small amount of blood; proiomenorrhea - when menstrual bleeding can occur every 20 to 25 days; polimenorrhea - when bleeding repeats every 15 days; and opsomenorrhea - when the interval between bleedings is 35 to 40 days.. ^[[40]](#endnote-40)^

**• Metabolic and Cardiovascular Changes in Menopause:**

With the reduction of estrogen production in menopause, there are significant morphophysiological and cellular changes that modify the secretion of the hormone Leptin, which is the hormone of satiety and energy balance. This hormone is involved in various regulatory processes in the female body, such as angiogenesis, the inflammatory process, lipid metabolism, and signaling for other hormones according to the literature. Leptin is closely linked to obesity.^[[41]](#endnote-41)^.

Estrogen regulates liver function by acting on genes that control metabolism. During the reproductive phase and menopause, changes in lipid profiles occur, promoting the onset of cardiovascular disorders. Glucose intolerance in menopause is also described in the literature as another issue related to estrogen deficiency. This is because the development of insulin resistance and diabetes occurs due to excess abdominal fat.^[[42]](#endnote-42)^. After menopause, women develop a more atherogenic lipid profile, with an increase in LDL levels and a decrease in HDL levels, becoming a potential vascular risk factor. ^[[43]](#endnote-43)^.

The main changes identified in the lipid profile during menopause are an increase in triglyceride levels, especially LDL-C, and a decrease in HDL-C. The beneficial effects of estrogen on the serum lipid profile can be attributed to the regulation of hepatic expression of genes involved in the metabolism of serum lipoproteins, such as lipoprotein A. In addition to its antioxidant property, its effect can be attributed to its phenolic structure, which scavenges free radicals, attenuating their deleterious effects on cellular metabolism, such as lipid peroxidation.^42^.

Estrogen has a protective effect on cardiomyocytes, inhibiting apoptosis and maintaining the integrity of collagen fibers in the heart, preventing cardiac fibrotic processes. In menopause, studies indicate a relationship between the increase in heart diseases after the age of 50, such as Acute Myocardial Infarction (AMI) and Stroke (CVA), in women.^[[44]](#endnote-44)^.

Estrogens promote vasodilation, reduce homocysteine levels, have a neutral effect on C-reactive protein, and decrease fibrinogen levels. ^[[45]](#endnote-45)^.

- **Changes in bone metabolism during Menopause:**

Osteoblasts have estrogen receptors, which are responsible for bone resorption; estrogen is an important regulator of this activity. Hypoestrogenism interferes with the action of osteoblasts and increases the activity of osteoclasts, reducing calcitonin levels, resulting in bone loss and osteoporosis.^[[46]](#endnote-46)^.

Sex hormones play an important role in bone growth and the maintenance of peak bone mass. Studies show evidence that the decrease or absence of estrogens progressively leads to a reduction in bone mass. In young women undergoing oophorectomy or early menopause, an accelerated bone loss is observed with a faster onset of osteoporosis.^[[47]](#endnote-47)^.

Estrogen receptors and selective estrogen receptor modulators (SERMs) act in the phase of inhibition and resorption. These, in turn, promote the apoptosis of osteoclasts through non-genomic mechanisms via the cell membrane receptor. It is possible that the binding of estrogens to the receptor present in osteoblasts indirectly regulates the function of osteoclasts, facilitating a cell-to-cell interaction. The binding of cytokines to osteoblastic receptors releases soluble factors that act directly on the osteoclastic lineage, regulating their recruitment and activity. Estrogens appear to inhibit the release of osteoclast-stimulating factors or increase their inhibitory factors.^39^.

Studies indicate that estrogen plays a role in bone remodeling by controlling the synthesis of cytokines by osteoblasts and osteoclasts in an autocrine or paracrine manner. ^39.^

- **Changes in the Central Nervous System in Menopause**

The hypothalamus, amygdala, hippocampus, locus coeruleus, and frontal lobe have estrogen receptors, so they are directly influenced by the decline in estrogen. Consequently, women in this phase experience changes in cognition, mood, memory, and sleep quality, impacting their overall quality of life. These alterations tend to trigger neurodegenerative disorders due to functional losses resulting from decreased circulating estrogen affecting specific brain areas. Additionally, they affect levels of serotonin, norepinephrine, acetylcholine, and dopamine – crucial neurotransmitters that can compromise synaptic connections. ^31^

Evidence suggests that elevated cortisol levels, combined with physical and psychological factors, along with hypoestrogenism, accelerate neuronal degeneration. Thus, the neuroinflammatory process during menopause may contribute to the onset or progression of neurodegenerative diseases in women, such as Parkinson's disease and Alzheimer's disease. ^[[48]](#endnote-48)^

**• Changes in the Integumentary System:**

The decrease in estrogen causes a progressive loss of cutaneous collagen, leading to a reduction in skin tone and accelerated wrinkle formation. Another factor is body water retention, directly influenced by female hormones that increase the reserve of glycosaminoglycans and collagen, improving skin elasticity. As women age, some may experience drier, thinner skin and delayed wound healing. ^48^.

Due to hypoestrogenism, the degree of thinning that occurs in aging skin may be directly related to estrogen receptors present in the skin. The main consequences on the skin and its appendages resulting from hypoestrogenism include an aged appearance with loss of normal texture, decreased elasticity, dryness, nails exhibiting slowed growth, and hair showing reduced growth rates with shortening of the anagen phase. ^39^.

• **Urogenital Changes in Menopause:**

Hypoestrogenism affects the normal structure and function of genital tissues, contributing to the loss of mucosa elasticity and inducing the fusion and hyalinization of collagen fibers, as well as the fragmentation of elastin fibers. There is a decrease in vaginal mucosa hydration in the dermal layer, with a reduction in mucopolysaccharides and intercellular hyaluronic acid, resulting in a thin stratified epithelium with only basal and parabasal layers. ^[[49]](#endnote-49)^.

The genital epithelium becomes thinner, paler, and drier, leading to potential vaginal restriction and shortening. The mucosa may become less elastic, gradually losing rugosity, and experiencing changes in vaginal microbiota, along with reduced blood flow. In cases of severe atrophy, the vestibule and vagina's surface can become friable, with petechiae and ulcerations, easily bleeding, and may even exhibit stenosis and narrowing of the vaginal fornices. The discomfort associated with vulvovaginal atrophy can significantly impact overall health and quality of life (figure 2), although patients without sexual activity may go through this period without experiencing most of the mentioned symptoms. ^49^, ^17^.

The discomfort associated with vulvovaginal atrophy (Figure 2) can have a significant impact on overall health and quality of life; however, patients without sexual activity may go through this period without experiencing most of the mentioned symptoms^17^.

Hypoestrogenism is the main factor for a condition called Genitourinary Syndrome (GS), which includes dryness, burning, irritation, sexual symptoms like discomfort or pain, and affected sexual function, as well as complaints of incontinence, dysuria, straining (slow and painful urination), and recurrent urinary tract infections (UTIs). The symptomatic picture corresponds to the loss of collagen and adipocytes, making the urogenital epithelium thinner (more vulnerable to trauma) and less elastic, with reduced blood flow, attenuation of gland function leading to reduced lubrication and irritation, changes in vaginal flora making it more susceptible to pathogenic bacteria, and increased susceptibility to vaginal infections, bacterial vaginosis, and sexually transmitted infections (STIs). ^17^, ^49^.

The common embryonic origin during fetal development of the lower urinary tract and external genitalia explains the pathophysiological effects of estrogen deficiency on these anatomical structures. Evidence has shown that estrogen receptors (alpha and beta) are present in the vagina, vulvar vestibule, urethra, bladder trigone, and in autonomous and sensory neurons in the vagina and vulva. While both receptors can be found in premenopausal women, only estrogen receptor-b can be detected in postmenopausal women. ^[[50]](#endnote-50)^.

Irritation and trauma during sexual intercourse result from the hypoestrogenic environment of urogenital tissue and reflect changes in the thickness of the vaginal and subepithelial epithelium, smooth muscle atrophy, reduced blood flow to the vaginal area, and tissue elasticity loss (reduction in collagen, elastin, and hyaluronic acid concentration). The decrease in glycogen concentration in vaginal cells leads to alterations in the vaginal microbiome (fewer lactobacilli) and an increase in vaginal pH. The combined effect of these pathophysiological mechanisms results in changes in the urogenital system, mainly as alterations in vaginal mucosa (paleness and fragility) and discharge, changes in the consistency of the vaginal microbiome, decreased pubic and subcutaneous hair, labial fat loss, and reduced size of the vestibular bulbs. In the lower urinary tract, they affect the capacity and contractile ability of the bladder, urethral sphincter, and functionality of the pelvic floor muscles. ^17^

A reduction in estrogen levels causes a decrease in ureteral pressure, leading to urinary incontinence, initially on exertion and with progressive worsening. This symptomatology often interferes with a woman's sexual activity, reducing libido and self-esteem. ^45^.

Urinary incontinence is one of the main problems affecting postmenopausal women, causing significant social, psychological, and economic impact, often being disabling. Its frequency is influenced by various factors such as parity, age, diagnostic criteria, among others. ^39^.

Urogenital changes during menopause, in addition to vulvovaginal atrophy, include stress incontinence, urgency incontinence, and recurrent urinary tract infections (UTIs). These conditions result from urogenital aging due to decreased estrogen levels with epithelial and subepithelial atrophy and connective tissue degeneration. ^[[51]](#endnote-51)^.

As the urogenital mucosa becomes thinner and less cellular, it also reduces glycogen production, resulting in a decrease in lactobacillus colonization, alkalization of vaginal pH, and an increased susceptibility to vulvovaginal infections and UTIs. Estrogen deficiency and low collagen content contribute to decreased pelvic muscle tone, with a consequent increased risk of urogenital prolapse. Existing estrogen and progesterone receptors in the lower urinary tract corroborate with the elements responsible for urinary continence, such as urethral mucosa, alpha-adrenergic receptors of the urethra, pelvic floor collagen, and peri-urethral vessels with the effects of estrogen deficiency in women at this stage. ^51^

Another important factor demonstrated in the literature is collagen tissue, which accounts for 30% of the total protein in the body and is essential for urinary continence, as it participates in the formation of ligaments and fasciae responsible for supporting and suspending pelvic organs. Estrogen's hormonal influence on the bladder's connective tissue can increase collagen due to changes in the extracellular matrix of the bladder's lamina propria. The deposition of collagen fibers in the detrusor alters bladder contractility, leading to urinary symptoms. The loss of bladder compliance can determine various urinary changes such as urgency, incontinence, vesicoureteral reflux, hydronephrosis, among others. ^51^

. In the bladder, the loss of estrogens causes a decrease in detrusor muscle sensitivity, resulting in increased urinary frequency, nocturia, and incontinence. This can be either stress or urgency incontinence, usually being mixed and reported in 10 to 35% of postmenopausal women. The reduction in the thickness of the mucosa and submucosa, as well as the content of collagen and elastin in the urethra, decreases the average closing pressure. ^[[52]](#endnote-52)^.

The prevalence of urgency incontinence increases with the number of years postmenopause. In contrast, stress incontinence is more frequent in the perimenopausal years, with no marked increase afterward. Coital incontinence is described in 11% of incontinent women. Some explanations identified in the literature for this urinary condition include decreased blood flow to the urethral tissues, causing sphincteric fibrosis and loss of resistance, and thinning of the urethral mucosa compromising the urethra's ability to form a continent mucosal seal through coaptation^51, 52^.

It is proven that urinary tract infection and asymptomatic bacteriuria are more frequent in postmenopause; they result from changes in vaginal flora (reduction of lactobacilli and increased pH) and likely local immunodeficiency with greater bacterial adherence to the urothelium. The existing hormonal receptors, both estrogen and progesterone, in the lower urinary tract contribute to these elements responsible for urinary continence, such as urethral mucosa, alpha-adrenergic receptors of the urethra, pelvic floor collagen, and peri-urethral vessels, with the effects of estrogen deficiency in women at this stage^39^.

Another important factor demonstrated in the literature is collagen tissue, which accounts for 30% of the total protein in the body and is essential for urinary continence as it participates in the formation of ligaments and fascia responsible for supporting and suspending pelvic organs. Estrogen's hormonal influence on the bladder's connective tissue can increase collagen due to changes in the extracellular matrix of the bladder's lamina propria. The deposition of collagen fibers in the detrusor alters bladder contractility, leading to urinary symptoms. It is known that the loss of vesical compliance can determine various urinary changes such as urgency, incontinence, vesicoureteral reflux, hydronephrosis, among others^39^.

Regarding alpha-adrenergic activity in the urinary tract and pelvic floor, estrogens increase the number and sensitivity of alpha-adrenergic receptors, as well as the membrane potential, and block the extraneuronal reuptake of catecholamines, thus increasing the tone of the urethral sphincter. The vascular network of the urethra accounts for about a third of urethral pressure and is influenced by estrogen, especially in its proximal portion, hence its relevance to maintaining urinary continence.^39^.

## 1.4. Menopause Diagnosis:

According to SOBRAC (Brazilian Society of Cardiology)^39^, the investigation of the patient begins with a thorough medical history and gynecological examination, which will provide essential information for a correct diagnosis.

According to its guidelines, the medical history should include the onset of symptoms, their duration, intensity, and any associated conditions that may have precipitated the clinical picture. In clinical complaints, there may be references to hot flashes, insomnia, irritability, arthralgia, myalgia, palpitations, decreased memory and interest in routine activities, decreased libido, dyspareunia, asthenia, and genitourinary symptoms related to mucosal atrophy. The age of menarche and the date of the last menstruation/menopause are important, as well as the manner in which each occurred. The presence of menstrual irregularities should also be noted ^53^.

Inquire about the use of medications (diuretics, alpha and beta-adrenergic blockers, and drugs with cholinergic action), especially in older patients, as many of them influence the physiological activity of the bladder and urethra. This fact may lead women to seek their gynecologist due to genitourinary complaintsParte superior do formulário

^39^.

Evaluate personal, family, menstrual, sexual, and obstetric history; the woman's sexual orientation is important information and should be considered without prejudice. Investigate the contraceptive methods used, the woman's vulnerability to STD/AIDS, and the performance of preventive tests for cervical cancer and early detection of breast cancer. Data about gastrointestinal function indicate the presence or risk of developing diseases ^53^.

Check dietary habits (fiber intake, fats, foods rich in calcium, and simple carbohydrates), physical activities (type, regularity, and duration), as well as the presence of concomitant pathologies, allergies, and personal issues related to romantic or family relationships. Regarding family history, inquire about diabetes mellitus, hypertension, cardiovascular diseases, gastrointestinal conditions, osteoporosis, as well as breast, uterine (cervix or endometrium), ovarian, or other types of cancer^53^.

In the general physical examination, the necessary anthropometric measurements include checking the weight and height to calculate the Body Mass Index (BMI) (weight/height²). Additionally, measure blood pressure and assess abdominal circumference (greater than 80 cm in women) ^53^.

In the gynecological examination, it begins with the assessment of the breasts, involving careful inspection and palpation, including the armpits and lymph nodes, to detect any changes. Following this, a thorough inspection of the vulva is conducted, with attention to the occurrence of alterations^39^.

During the speculum examination, we should assess signs of atrophy due to hypoestrogenism, such as thin and friable mucosa, urethral caruncle, stenosis of the vaginal fornices, and decreased rugosity. The integrity of the pelvic floor musculature must be examined. It is essential to investigate the strength of voluntary contraction of the pelvic floor muscles, the tone of the anal sphincter, and any potential genital descent. The function of the pelvic floor can be evaluated through a digital exam or with perineometers. Urodynamic examination is highly relevant in diagnostics, as it allows the recording of vesical, abdominal, and urethral pressures during bladder filling and emptying^39^.

The complementary exams for monitoring women during perimenopause/menopause include: laboratory assessment, mammography, breast ultrasound, transvaginal ultrasound, cervical cancer screening (Pap smear), and bone densitometry^53^.

In postmenopause, laboratory diagnosis shows a roughly 10 to 15 times increase in FSH, while LH increases 3 to 5 times (Figure 3). Estradiol, decreasing by up to 80%, is gradually replaced by estrone, which predominates in postmenopause. Serum estradiol is then the result of the peripheral conversion of androgens produced by the ovaries (stroma) and adrenal glands into estrone. This conversion occurs in adipose tissue, liver, muscles, kidneys, and probably in the skin. The FSH level is sufficient for the diagnosis of ovarian hypofunction or failure when the result is higher than 40 mIU/ml^[[53]](#endnote-53)^.


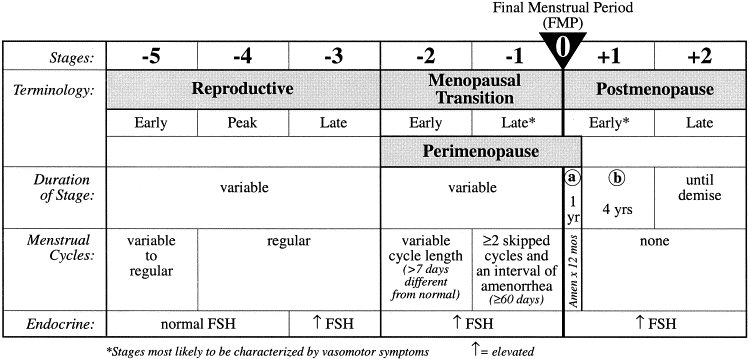


Figure 3 – Stages of normal reproductive aging in women^[[54]](#endnote-54)^

**1.5. Menopause Treatments.**

### 1.5.1. Hormone Therapy:

Several treatments have been found in the literature for menopausal women to alleviate GSM (Genitourinary Syndrome of Menopause). Among them are estrogen hormone replacement (local or systemic), the use of isoflavones, lubricants, radiofrequency, and laser application. All identified treatment options, whether isolated therapy with estriol, lasers, or a combination of both treatments, have resulted in improved vaginal health and symptoms of atrophy, whether the symptoms are mild or severe.

The objective of the treatment for atrophic vulvovaginitis is to alleviate symptoms related to one's sexual life. Currently, scientific evidence^[[55]](#endnote-55)^ The use of Hormone Therapy (HT) is recommended in four situations: presence of vasomotor symptoms, genitourinary syndrome of menopause, prevention of bone mass loss, and early menopause.

It is recommended that before starting Hormone Therapy (HT), a thorough clinical assessment be conducted to assess the risks and benefits and determine the best route of hormonal administrationl^55.^ It is important to emphasize that, in addition to assessing a woman's health, understanding how she perceives her health conditions is a crucial factor for the adoption of preventive methods and well-being promotion, enhancing the quality of life during this phase^[[56]](#endnote-56)^.

Some contraindications for Hormone Therapy (HT) have been identified by researchers, including a personal history of breast cancer, critical liver or kidney insufficiency, a history of acute and recurrent thromboembolism. Studies indicate that HT may increase the risks of cardiovascular diseases, especially when associated with conjugated equine estrogens and medroxyprogesterone acetate^[[57]](#endnote-57)^.

According to research, there are indications that Hormone Replacement Therapy (HRT) influences plasma cell lipoprotein concentrations, thereby reducing the risks of coronary diseases in women. The chances of developing colon cancer in patients who underwent therapy at some point in life are decreased by 20%, and by 34% in regular users. Other benefits include the prevention of osteoporotic fractures, verbal memory, reasoning, motor speed, and lower susceptibility to urological infections^[[58]](#endnote-58)^.

In the last Brazilian multidisciplinary consensus on assistance for women in menopause ^39^ the recommended hormonal replacement therapy includes the use of estrogens, progestogens, and, occasionally, androgens. Hysterectomized patients do not require progestogen use. In this situation, estrogens can be used alone and continuously. In cases of patients treated for endometriosis and early-stage endometrial cancer, the use of estrogens is most appropriate. In women with an intact uterus, estrogens are used in combination with progestogens. To prevent bleeding observed in these circumstances, estrogens can be used daily and continuously in combination with reduced doses of progestogens, in a scheme called continuous combined therapy.

It is important to emphasize that, in addition to assessing a woman's health, understanding how she perceives her health conditions is a crucial factor for the adoption of preventive methods and well-being promotion, enhancing the quality of life during this phase.It is important to emphasize that, in addition to assessing a woman's health, understanding how she perceives her health conditions is a crucial factor for the adoption of preventive methods and well-being promotion, enhancing the quality of life during this phase.It is important to emphasize that, in addition to assessing a woman's health, understanding how she perceives her health conditions is a crucial factor for the adoption of preventive methods and well-being promotion, enhancing the quality of life during this phase.It is important to emphasize that, in addition to assessing a woman's health, understanding how she perceives her health conditions is a crucial factor for the adoption of preventive methods and well-being promotion, enhancing the quality of life during this phase.It is important to emphasize that, in addition to assessing a woman's health, understanding how she perceives her health conditions is a crucial factor for the adoption of preventive methods and well-being promotion, enhancing the quality of life during this phase.

In this therapeutic regimen, the continuous use of progestogens leads to endometrial atrophy over time, resulting in amenorrhea in most users. A variant of the continuous combined scheme is represented by the regimen in which estrogens are used continuously, and progestogens are employed in cycles of three days on and three days off, in a therapeutic scheme called intermittent combined therapy, which, similarly over time, leads to endometrial atrophy and amenorrhea^[[59]](#endnote-59)^.

Estrogens are used in Hormone Replacement Therapy (HRT) at doses capable of relieving vasomotor symptoms, preventing or treating urogenital atrophy and osteoporosis. They can be administered orally or non-orally. Non-oral routes include vaginal, nasal, transdermal (patch or gel), and subcutaneous implants (Table 1). The vaginal route is not indicated for systemic hormone replacement but can be used in the prevention or treatment of urogenital trophic disorders.

Tabela 1- Classificação e apresentação dos estrógenos usados em THM^59^


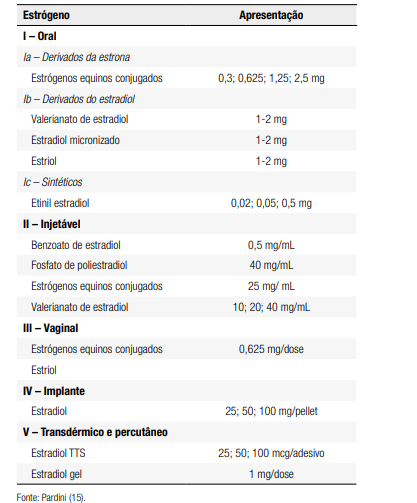


### 1.5.2. Non-Hormonal Therapies:

Non-pharmacological approaches are beneficial, especially in women with contraindications to hormone use or those who prefer not to use them. However, they can also be used as adjuvant or substitute therapy for any patient^49^.

Among the options raised in the literature, we have the following:

1. **Phytotherapy:**

An important therapeutic option in menopause, especially in the treatment of associated symptoms, herbal remedies with stimulating properties on specific hormonal receptors (beta receptors) exist. This improves the clinical manifestations presented. The significant advantage of these herbal remedies is their highly selective action, being considered Selective Estrogen Receptor Modulators (SERMs). This results in very low rates of side effects for such substances^53^.

The main herbal remedies used in menopause are phytoestrogens, which, due to their estrogen-simile action, also exert antiestrogenic effects. The extrinsic biological activity of these herbal remedies stems from their chemical composition, which contains heterocyclic phenolic rings similar to natural and synthetic estrogens compatible with beta-estrogen receptors^53^.

Among them, the most commonly used for menopause are Glycine Max, Trifolium pratense, and Cimicifuga racemosa, although there are many other herbal remedies for this purpose. However, it is necessary to check for dysbiosis, characterized by poor digestion, poor food absorption, constipation, and intestinal gas. If this occurs, the dietary pattern of women using this therapy should be adjusted^53^.

1. **Lubricants/Moisturizers:**

Vaginal lubricants and moisturizers can be used continuously for mild symptoms and primarily improve dryness and dyspareunia. However, they may alter the vaginal microbiota and increase the risk of infection or cause vaginal discharge. In general, these products are effective and well-tolerated^[[60]](#endnote-60)^.

A vaginal moisturizer composed of polyacrylic acid, a calcium salt linked to divinyl glycol, which, in an acidic environment, releases calcium and can absorb 60 times its weight in water. Due to this absorption capacity, it provides a therapeutic effect of vaginal hydration and lubrication, improving symptoms of urogenital atrophy when compared to estrogen^[[61]](#endnote-61)^. There is evidence that polyacrylic acid associated with estriol has shown an effect in improving urogenital atrophy, though without changing the vaginal pH^[[62]](#endnote-62)^..

It is important to emphasize that studies indicate that neither lubricants nor polyacrylic gel alter vaginal cytology morphology, improve pH, nor reduce lower urinary tract symptoms such as dysuria and urinary urgency. The use of lubricants during sexual intercourse can help reduce irritation and mechanical trauma caused by tissue friction.

The regular use of long-acting moisturizers reduces vaginal pH, bringing it to premenopausal levels, and hydrates the vaginal mucosa, but does not improve the vaginal maturation index^17^. In Brazil, some products based on synthetic polymers containing polyacrylic acid are available in the market in tubes with 30g of gel. Other moisturizers based on hydrolyzed hyaluronic acid are presented in tubes with 24g of gel. They hydrate and restore moisture for 72 hours, hence considered long-lasting. All moisturizers are for vaginal use exclusively and should be used everythree days^[[63]](#endnote-63)^.

1. **Ospemifene:**

It is a Selective Estrogen Receptor Modulator (SERM) that binds to ERα and ERβ receptors, with slightly greater affinity for the ERα receptor, exerting agonistic effects on the vaginal epithelium with minimal endometrial effectl^[[64]](#endnote-64)^. It is the only non-systemic therapeutic option for GSM approved by the FDA, acting as a selective estrogen receptor modulator. This drug (at a dose of 60mg) has shown to be effective and safe, restoring vaginal pH and significantly and consistently reducing symptoms of dyspareunia and vaginal dryness^1, 4^. It is not yet commercially available in Brazil ^63^.

1. **Radiofrequency (RF):**

Transcutaneous monopolar RF is the most used in gynecology. It operates based on two electrodes: one passive, in contact with the patient, and a second one that emits RF to the first through the body. The goal is to promote heating of the vaginal epithelium to approximately 40-45°C for 3-5 minutes per application area, totaling 25-30 minutes per session. These sessions can be repeated at intervals of 4 to 6 weeks. Histologically, this leads to epithelial regeneration, including secretion, absorption, lubrication, and structural integrity and thickness^49.^

Another study on RF in menopause suggests that the treatment is well-tolerated by women and leads to a significant improvement in the vaginal microenvironment; therefore, radiofrequency can be used to treat vaginal symptoms of GSM. The therapy restored vaginal balance, as would normally be expected with sufficient estrogen levels. The predominance of Lactobacillus species and the acidic pH of vaginal fluid achieved after radiofrequency therapy may protect postmenopausal women from vaginal infections, inflammation, and urogenital tract infections with limitations in the sample size^[[65]](#endnote-65)^.

## 1.6. Laser:

Laser therapy has been used in GSM in menopause. In general, the mechanism of action involves applying heat to the tissue, which generates controlled thermal damage, stimulating fibroblasts to produce neocollagenesis. The increase in temperature determines collagen remodeling, new collagen formation, and overall improvement in the stiffness and elasticity of the vaginal tissue. It is known that the loss of vaginal rugosity and elasticity, related to menopause, is due to the breakdown of collagen supporting the vagina's epithelium ^63^.

The term LASER is an acronym for Light Amplification by Stimulated Emission of Radiation. The laser beam is formed by concentrated particles of light (photons) emitted in the form of a continuous beam. To achieve this effect, it is necessary to stimulate atoms of some material to emit photons. This light is channeled with the help of mirrors to form a beam ^[[66]](#endnote-66)^.

Light is composed of particles and propagates in waves. All electromagnetic waves have general characteristics of wavelength, amplitude, and frequency. The wavelength is the distance from one peak to another, amplitude represents the height of the peak (including the valley), and the number of peaks per unit of time (seconds) – frequency is measured in Hertz (Hz) or cycles per second. The laser beam has three characteristics that differentiate it from ordinary light ^66^:

Coherence - all wavelengths are in phase with each other, meaning the peaks and valleys of the waves occur simultaneously.

Collimation - all wavelengths are parallel to each other, with little or no divergence.

Monochromaticity - all have the same frequency or wavelength and, therefore, the same color.

The medium used to create the laser (solid, gas, or liquid) determines the wavelength and color of the beam and its properties. The laser power is regulated by the amount of energy spent to stimulate light emission. Thus, each wavelength reacts differently at the applied sites, and energy density, which is the amount of energy per unit area, also influences the effect^[[67]](#endnote-67)^.

The laser interacts with living tissues through optical processes of reflection, transmission, scattering, and absorption. When incident on biological tissue, only the absorbed part of the light will act effectively. The absorbed energy is measured in Joules/cm2 and is known as energy density or fluence. Laser light absorption depends on the amount of chromophore present in the tissue and the match between the wavelength used and the absorption characteristics of that chromophore. Once absorbed, light can cause three basic effects: photothermal, photochemical, and photomechanical ^[[68]](#endnote-68)^.

Absorption occurs when the emitted electromagnetic wave is absorbed by the chromophores located in the tissues. In this sense, the interaction of different wavelengths with their respective chromophores, also located in different types of biological tissues, becomes clearer as shown in the figure below:


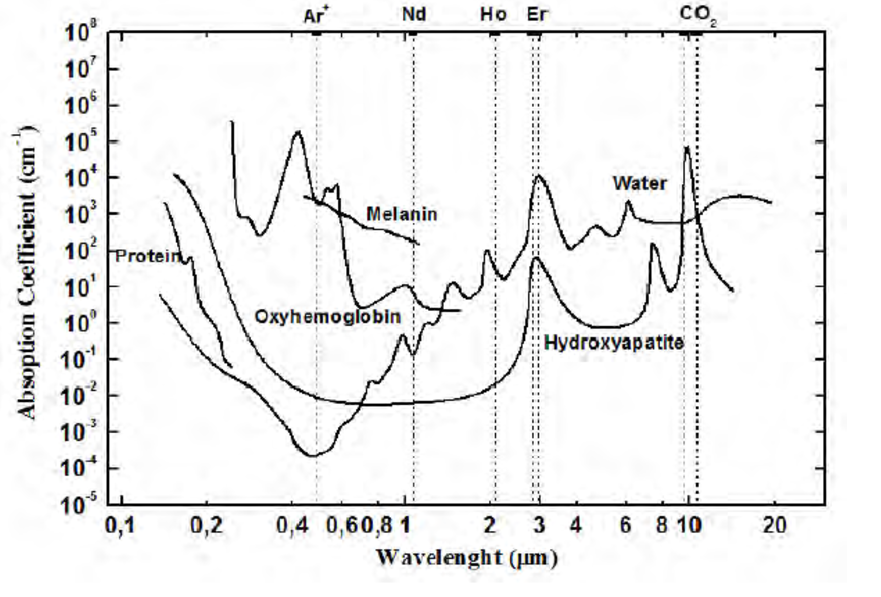
Parte superior do formulário

**Figure 4 - Main Chromophores of Biological Tissues ^[[69]](#endnote-69)^**

The photothermal effect occurs when the chromophore absorbs energy with the corresponding wavelength, and the light energy converts into heat capable of destroying the targeted area. In addition to the laser effects, the specific characteristics of each tissue are also important, such as tissue absorption coefficient, scattering coefficient, tissue refractive index, cell types, blood perfusion, thermal conduction, tissue oxygenation, and inflammation, infection, or necrosis ^68^.

The depth of laser energy penetration into tissues depends on absorption and scattering. The scattering of laser energy is inversely proportional to the wavelength. The longer the wavelength, the deeper the penetration of laser energy. Wavelengths between 300 and 400 nm scatter more and penetrate less. Wavelengths between 1,000 and 1,200 nm scatter less and penetrate more. However, energies with wavelengths in the mid and upper infrared range of the electromagnetic spectrum are absorbed superficially, as the main chromophore for this wavelength range is water present in the tissue Parte superior do formulário

^68^.

For pulsed laser emission, it is necessary to consider temporal factors such as the form of light emission (continuous or pulsed), the repetition rate, and the pulse width ^66^.

There is evidence that the results obtained with this type of technique are very promising in reducing the severity of GSM symptoms and improving the quality of life. However, studies indicate that its impact and long-term consequences still require more randomized studies with a larger sample size for secure recommendation in current clinical practice.

**1.6.1. High-Power Laser Platforms:**

According to the literature, there are two types of lasers for vaginal use: erbium laser and CO2 laser, which are used on an outpatient basis. The procedures typically last 20 to 30 minutes and do not require anesthesia, except in cases of vulva and perineum treatment ^[[70]](#endnote-70)^.

The CO2 laser is indicated as the gold standard in dermatology for multiple mucosal and skin lesions. It is commonly applied in gynecology for the ablation of cervical lesions (such as warts). This technique uses light rays with a wavelength of 10,600 µm from a CO2 gas medium, strongly absorbed by water. This allows penetration into tissues essentially according to their water content and the intensity per unit of time of the applied pulse. It has an optical penetration of up to 50µm and a horizontal extent of 10 mm.

It produces thermal coagulation up to 1mm in depth, with a residual thermal damage zone of 100-150µm in pulses less than 1 millisecond. The applied energy density is directly related to the depth of ablation, and in less than 1 millisecond, it can penetrate about 20 to 30 µm in a well-limited diameter, thus avoiding undesirable effects in the adjacent area. The device parameters can be adjusted by the operator, but there are protocols defined by the manufacturers. The most well-known CO2 laser platform models in Brazil are from Alma Lasers (Keisarya, Israel) and Deka Laser (Florence,Italy) ^67^.

The fractional laser creates a pattern of microscopic ablative zones surrounded by adjacent normal zones. This microablative therapy results in cellular vaporization and induces irreversible coagulation and protein denaturation at temperatures between 50 and 65°C. As part of the wound healing process, production of heat shock proteins (HSP), especially HSP47, begins at temperatures above 45°C, stimulating fibroblasts for collagen synthesis ^67, 68^.

The connective tissue of the lamina propria is primarily composed of proteoglycans, which are macromolecules linked to long chains of hyaluronic acid through proteins and have the ability to retain large amounts of water. The hydration level of the lamina propria depends on these proteoglycans^67, 68^.

The rich water content in the extracellular matrix contributes to the mucosal turgidity, cooperating with the support function of well-structured collagen. The presence of water molecules facilitates mucosal metabolism, easier transport of metabolites and nutrients from capillaries to tissues, and the drainage of residual substances from tissues to blood vessels and lymphatics^67, 68^.

If the fundamental substance is poor or lacks much water, the epithelium will not receive the necessary nutrition for proper trophism. These phenomena form the basis of the thermal effect caused by the laser on the vaginal mucosa^67, 68^.

The vaginal erbium laser (VEL) is a non-ablative solid-state laser in aluminum yttrium garnet crystal (Er:YAG). This procedure is widely used in the treatment of dermatological and dental lesions, utilizing a solid medium for emitting continuous rays with a wavelength near the absorptive peak of water - 2940 nm. It has a high affinity for tissues rich in water molecules (about 16 times higher than CO2 laser) and limited penetration between 1 to 3 μm, allowing for a much more precise ablation capability with minimal adjacent thermal damage^68^.

This method does not have the coagulation properties of the previous one, so the likelihood of bleeding during its use is higher. However, it is associated with faster recovery and less discomfort, erythema, and edema. Recently, this method has also been tested in a non-ablative manner with rapid sequences of less intense and intermittent pulses, leading to more discreet increases in mucosal temperature and avoiding excessive ablation ^68^.

The erbium laser has an optical penetration of 3 to 5 µm and a penetration depth of 200-300 µm, acts through thermal diffusion, and does not cause tissue ablation. The application parameters are determined by the device and based on previous clinical studies. The platform has a low-fluence sequence and erbium pulses, which distribute heating up to 100 μm deep in the mucosal surface, producing a deep thermal effect without tissue ablation or carbonization, avoiding the risk of perforation with accidental injuries to the urethra, bladder, or rectum Parte superior do formulário

^[[71]](#endnote-71)^.

The most well-known platform is the XS Fotona Smooth™, developed by Fotona (Ljubljana, Slovenia). It has a wavelength of 2940 µm and absorbs fifteen times more water than the CO2 laser. Wallace^[[72]](#endnote-72)^ conducted a cost-effectiveness analysis of three therapies for genitourinary syndrome of menopause, including vaginal estrogen therapy, oral ospemifene therapy, and vaginal CO2 laser therapy, and demonstrated that all three treatment methods were considered cost-effective below the willingness-to-pay threshold of $50,000.00 per quality-adjusted life year for moderate dyspareunia. The cost-effectiveness ratio for vaginal CO2 laser therapy was $16,372.01, and the cost-effectiveness ratio for ospemifene therapy was $5,711.14. In conclusion, to establish vaginal laser therapy as a cost-effective treatment strategy for dyspareunia associated with genitourinary syndrome of menopause, health insurance coverage or a public health policy would be necessary, particularly in the case of Brazil.

**1.6.2. Effects of Laser on the Vaginal Epithelium**

The effects of high-power laser described in the literature depend on the characteristics of the target tissues, the regeneration time, and the results vary in each individual. Studies indicate that the regeneration process begins between 1 to 3 days with the development of granulation tissue with fibroblastic activity, increased cellular levels, and synthesis and storage of glycogen in the mucosa, as well as thickness and differentiation of the epithelium^18^.

The reorganization and proliferation of the extracellular matrix (collagen) of the lamina propria with the genesis of new papillae and penetrating capillaries allow adequate hydration, permeability, and metabolic support of the tissues. There is also an increase in the activity of lactobacilli with consequent restoration of premenopausal pH levels^[[73]](#endnote-73)^.

The figures below show the histology of the vaginal mucosa after the application of fractional CO2 laser, demonstrating its efficacy.


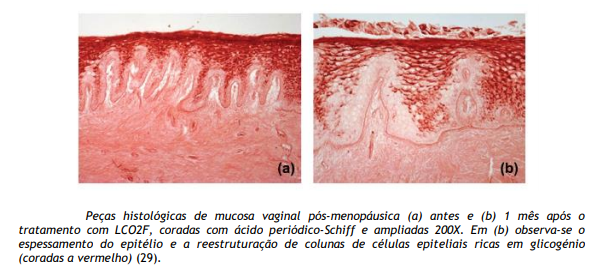


Figure 6 - Histology of the Vaginal Mucosa 1 Month After Fractional CO2 Laser (LCO2F)
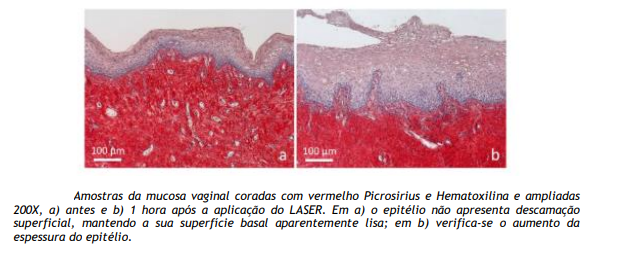


Figure 6 - Histology of the Vaginal Mucosa 1 Month After Fractional CO2 Laser (LCO2F) ^74^

1.6.3. **Contraindications for Vaginal Laser**

The main contraindications found in the literature for the use of vaginal laser are active or recent vaginal lesions, untreated genitourinary tract infections, abnormal uterine bleeding, a history of photosensitivity disorders or use of photosensitizing drugs, pelvic organ prolapse grade II-III (according to the Pelvic), bacterial or viral vaginal infection (HPV, Herpes), immunosuppression, chronic use of corticosteroids, scleroderma, extensive prior radiation therapy, burns in the area, collagen disorders, genital neoplasia, anticoagulant therapy, and patients with uncontrolled diabetes ^18, 23, 71.^

1.6.4. **Adverse Effects and Complications of Vaginal Laser Use**

The FDA (Federal Drug Administration) issued a statement on July 30, 2018, expressing concern about the marketing of lasers and energy-based devices to promote "vaginal rejuvenation." The FDA stated that there is not enough data to recommend this modality for optimizing sexual function and relieving symptoms related to genitourinary syndrome of menopause (GSM) in postmenopausal or postpartum women^[[74]](#endnote-74)^.

Adverse events associated with the use of laser and energy-based devices for "vaginal rejuvenation" have been reported to the MAUDE (Manufacturer and User Facility Device Experience) database, although the circumstances surrounding these events are not clear, highlighting the importance of clinical trials investigating the efficacy and safety profile of laser and energy-based devices for improving sexual function and treating symptoms related to GSM^75^. There is evidence pointing to a gap in the literature regarding the effectiveness and safety profile of vulvovaginal energy-based therapies, their indications, contraindications, maintenance regimens, comparison with current available treatments, and long-term benefits^[[75]](#endnote-75)^.

The effects described by studies, although few reported, resulted from mechanical trauma caused by the insertion of the probe or thermal ablation. Some effects that may occur immediately after each session or in the short term include mild vaginal bleeding, mild discomfort, nonspecific discharge, burning, and local pain, which in most cases resolve quickly and spontaneously. More severe, but quite rare, cases involve reported lacerations of the vaginal wall and severe bleeding. In the medium/long term, although very low, there is a risk of permanent tissue damage with the formation of adhesions, changes in sensitivity, dyspareunia, or recurrent infections^[[76]](#endnote-76)^.

**2. JUSTIFICATION:**

In this context, given the high prevalence of GSM and its impact on the overall health of postmenopausal women, as well as the high costs of CO2 and Erbium lasers for public health systems, the study of new non-pharmacological therapies that can be used as adjuvant or substitute therapy is necessary.

Laser therapy appears to be an effective, well-tolerated, painless method with few or no immediate or late complications, and it can be administered on an outpatient basis. The scientific community awaits further studies to confirm the effects of laser therapy used so far, to assess its effects on GSM, and to validate protocols. This study is justified by contributing more results on the efficacy of vaginal laser through photobiomodulation, pointing to a non-hormonal proposal for the treatment of Genitourinary Syndrome of Menopause with improvement in signs and symptoms and quality of life in this vital cycle of female senescence. It could become a treatment applied in the public health system for postmenopausal women, as there is currently no available treatment for genitourinary syndrome in the public network.

**3. HYPOTHESES:**

**3.1 Null hypothesis:** Vaginal photobiomodulation is not an effective option for the clinical treatment of genitourinary syndrome of menopause when compared to the placebo group.

**3.2 Alternative hypothesis:** Vaginal photobiomodulation is an effective option for the clinical treatment of genitourinary syndrome of menopause when compared to the placebo group.

**4. OBJECTIVES:**

**4.1. GENERAL:**

Evaluate the clinical response of participants with symptoms of postmenopausal genitourinary syndrome after vaginal (external) photobiomodulation.

**4.2. SPECIFIC:**

- Evaluate and compare the improvement of genitourinary syndrome symptoms in women treated with vaginal photobiomodulation compared to the placebo group using a Visual Analog Scale (VAS).
- Assess the signs of vulvovaginal atrophy through the parameters of the Vaginal Health Index (VHI).
- Evaluate and compare the effect on sexual function in women treated with vaginal photobiomodulation compared to the placebo group using the Female Sexual Function Index questionnaire (FSFI-6).
- Assess compare, and correlate the response of photobiomodulation to urinary complaints and the impact on quality of life using the International Consultation on Incontinence Questionnaire – Short Form (ICIQ-SF) and a 1-hour pad test.
- Evaluate and compare the response of vaginal photobiomodulation on pelvic floor strength compared to the placebo group using a vaginal dynamometer.
- Evaluate and compare the response of vaginal photobiomodulation on local temperature compared to the placebo group through measurement with a digital thermometer.

**5. METHODOLOGY:**

**5.1 Study Type:**

This is a controlled, randomized, double-blind clinical trial that will be submitted to the Research Ethics Committee of Universidade Nove de Julho (Uninove). Subsequently, the evaluation, selection, and proposed procedures of the study will be carried out at the Specialties Outpatient Clinic in the municipality of Vargem Grande Paulista from October 2022 to October 2023 (after ethics committee approval), upon acceptance and signing of the Informed Consent Form (ICF) – **APPENDIX I.**

**5.2 Recruitment:**

Female participants in the postmenopausal period with complaints associated with Genitourinary Syndrome of Menopause (GSM) who attend routine consultations at health units in the municipality of Vargem Grande Paulista will be selected. The study will also be promoted to 12 health units in the municipality (8 primary care services, 1 Psychosocial Care Center (CAPS), a Women's Reference Center (CRSM), and the Emergency Care Unit (PA), and a Physiotherapy Service). An online meeting will be held with the professionals of these units to publicize the "Menopause Outpatient Project" created for the care of women with complaints of genitourinary syndrome and urinary incontinence, where they will be referred to the research project according to the inclusion criteria of the study. An invitation (**APPENDIX A**) and an informative brochure about the research (**APPENDIX B)** and the Pad test (**APPENDIX C**) will be sent to the nurse responsible for the health unit, with the address of the Menopause Outpatient Clinic, the name of the nurse responsible for the research, contact phone, and the interview scheduling time. An agenda will be opened in the afternoon for participant appointments at the Menopause Outpatient Clinic, following all emergency measures for the prevention and control of the COVID-19 pandemic. This schedule (**APPENDIX D**) will also be made available to the nurses of the health units, in case they want to schedule participants for interviews.

**5.3. Inclusion and Exclusion Criteria:**

Women over 50 years of age in the postmenopausal period (amenorrhea for at least 12 months, without pathology involved), with one or more symptoms of GSM (dyspareunia, dryness, irritation, and vaginal burning and/or discomfort, vaginal and vulvar atrophy, vaginal and vulvar dryness, dysuria, polyuria, recurrent urinary infections), with complaints of stress and/or urgency urinary incontinence, and with a normal cervical cytology (Pap smear) performed in the last year, not using hormonal medication for the treatment of menopausal symptoms in the last 6 months, will be included. Participants with explicit refusal to participate in the research, a history of bilateral oophorectomy, and diseases such as recent myocardial infarction (MI), neoplasms, a history of thrombosis, liver failure, uncontrolled genital bleeding, genital condylomatosis, active genital herpes, and lower genital tract surgeries that prevent treatment will be excluded from the study.

**5.4. Methods for Evaluating GSM in Menopause:**

The literature presents various methods for evaluating GSM, which authors categorize into subjective and objective methods. It is important to measure GSM symptoms to compare and monitor pre- and post-treatment effects. Subjective methods include self-administered questionnaires that describe the participant's perception of clinical symptoms before and after the proposed treatment or regarding quality of life and sexual satisfaction. Emphasizing the patient's reported well-being as the main point of the outcome^18,^^[[77]](#endnote-77)^. The main validated questionnaires that will be used in this research are:

The FSFI (Female Sexual Function Index) was validated for Portuguese for the first time in 2007 by Hentschel and is widely used in Brazilian studies. The FSFI, or Female Sexual Function Index, is a specific and multidimensional questionnaire to assess female sexual response, covering 6 domains: desire, arousal, lubrication, orgasm, satisfaction, and pain, assessed in 19 questions about sexual activity in the last 4 weeks. It allows for a quick and multidimensional self-assessment of sexual function in women diagnosed with GSM ^[[78]](#endnote-78)^ (Annex II).

Option 0 (zero) indicates no sexual activity, and the other response options receive scores from 1 to 5 in ascending order related to the presence of the questioned function. The analysis will be conducted by combining the responses from the six domains: Desire items 1 and 2; Arousal items 3, 4, 5, and 6; Lubrication items 7, 8, 9, and 10; Orgasm items 11, 12, and 13; Satisfaction items 14, 15, and 16; Discomfort/Pain items 17, 18, and 19, as per the table below (Table 2). For domain scores, individual scores are summed, and the result is multiplied by the corresponding factor. To obtain the total scale score, the scores for each domain are summed, with higher scores indicating better sexual function. The threshold for sexual dysfunction is a score of 26 ^78.^ Based on the total score value, it would be possible to discriminate between populations at higher and lower risk of sexual dysfunction^[[79]](#endnote-79)^.

**Table 2 - FSFI Domain Scores**


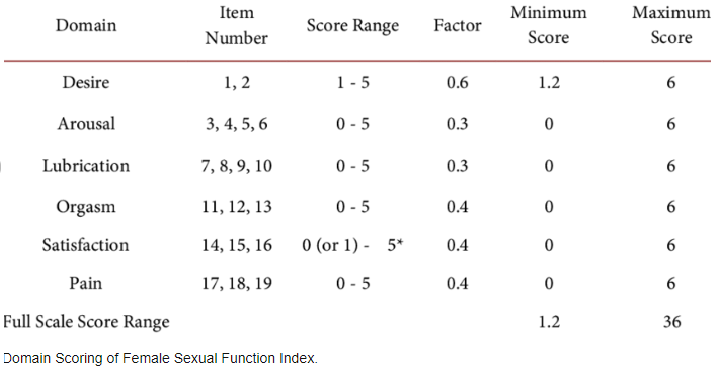


**Validation of the Female Sexual Function Index (FSFI) for use in the Portuguese language**^79^.

The International Consultation on Incontinence Questionnaire - Short Form (ICIQ-SF) is a simple, short, and self-administered questionnaire used to assess the impact of Urinary Incontinence (UI) on the quality of life and to qualify urinary loss in patients of both sexes. The ICIQ-SF was originally developed and validated in English by Avery et al ^[[80]](#endnote-80)^(ANNEX III).

In 2004, the ICIQ-SF was validated in Brazil with satisfactory reliability and is used as a method to monitor improvements in the quality of life post-treatment for urinary incontinence. It consists of four questions related to the frequency, severity of urinary loss, and its impact on the quality of life ^[[81]](#endnote-81)^. In addition, it includes a set of eight self-diagnostic items related to situations of UI experienced by individuals. This questionnaire has a score ranging from zero to 21 points, with zero indicating the absence of symptoms, and the higher the sum of points, the greater the impact and severity of the symptoms (Figure 10). The maximum sum of response values indicates a score of 21 points, corresponding to a high impact of UI on the individual's life^[[82]](#endnote-82)^.

To be considered incontinent, the obtained score must be ≥ 3, while the impact on quality of life is divided as follows: no impact (0 points); mild impact (1 to 3 points); moderate impact (4 to 6 points); severe impact (7 to 9 points); and very severe impact (10 or more points)^[[83]](#endnote-83)^.

**
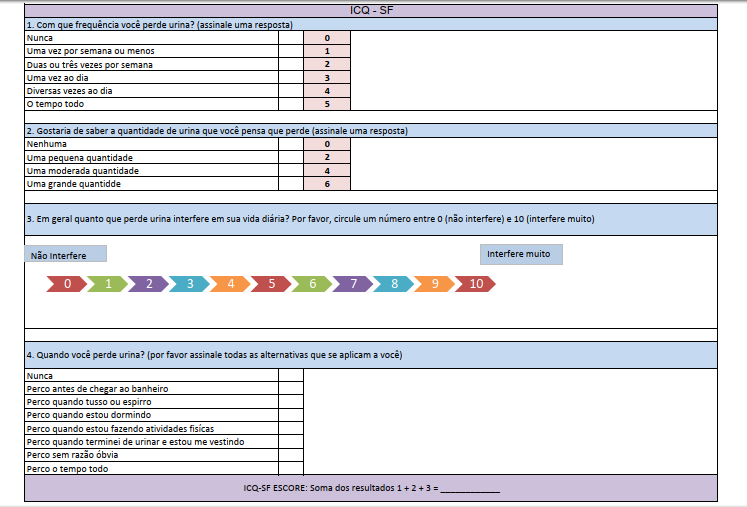
**

Figure 7 - ICQ-SF Questionnaire

Source: ICQ-SF^82^ - Adapted

The Vaginal Health Index Score (VHI Score) consists of a clinical analysis during the speculum examination of 5 parameters: elasticity, pH, mucosa appearance, moisture, and the presence of vaginal discharge. Each aspect assessed receives a score ranging from 1 to 5. The total score can vary from 5 to 25, with a diagnosis of Vulvovaginal Atrophy (VVA) considered when values are less than or equal to 15. The maximum score of 25 points indicates the absence of clinical signs of vulvovaginal atrophy (as shown below). This evaluation will be performed by a qualified professional during the physical examination.

**
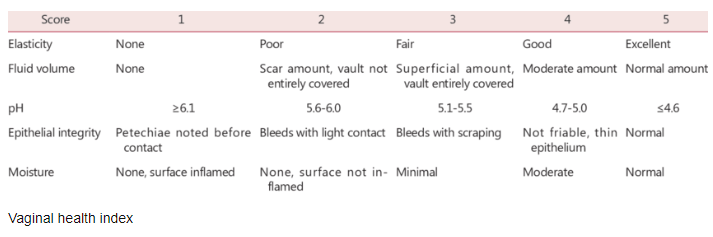
**

**Source: Bachmann, ET AL. 1995****^[[84]](#endnote-84)^ .**

Studies indicate that measuring vaginal pH is an alternative diagnostic test for confirming menopause and can be used in the office setting due to its cost-effectiveness and easy accessibility when compared to serum levels of FSH and estradiol. These studies demonstrated an increase in vaginal mucosa pH to around 6 in untreated menopause and a decrease to an average of 4.5 after appropriate estrogen treatment through any administration route. They then compared serum levels of FSH and estradiol with vaginal pH measurement in patients, concluding that both methods have similar sensitivity in identifying menopausal women or those with low serum estrogen levels. They concluded that pH measurement could be used independently for monitoring the introduction or dose adjustment of estrogen use in menopausal hormone therapy ^[[85]](#endnote-85),^ ^[[86]](#endnote-86)^.

The normal vaginal pH is acidic, and during the reproductive years, it should be maintained between 3.8 to 4.5. In the absence of infections, menopause is the main cause of an increase in vaginal pH. Some infections such as bacterial vaginosis, trichomoniasis, and group B streptococcus can also elevate the vaginal pH. According to Bachmann ^85^, circulating estrogen proliferates the cells of the vaginal epithelium, increasing the glycogen in mucosal cells, which stimulates the production of hydrogen peroxide and lactic acid, promoting the proliferation of lactobacilli and acidifying the vaginal pH. In menopause, mucosal atrophy leads to a decrease in glycogen, lactic acid, and lactobacilli, raising the vaginal pH to 5 to 7 ^[[87]](#endnote-87)^. The evaluation of vaginal pH will be performed using a pH indicator strip (pictured below), which, when inserted into the middle third of the vagina in contact with the mucosa, causes a change in the color of the strip. According to Roy ^86^, measurements in the vaginal fornix are avoided to prevent contact with mucus, blood, or semen, which can alter the reading. The nitrazine strip should remain in contact with the vaginal wall for 5 seconds for subsequent pH verification on the color scale provided by the manufacturer. Whe used the  MQuant Vaginal pH Indicator Strip test kit.

The Visual Analog Scale (VAS) consists of an instrument that assists in measuring the intensity of the participant's pain. It is recommended to assess the evolution of GSM symptoms during treatment and at each appointment more reliably. It will also be useful to analyze if the treatment is effective and which procedures have produced better results, as well as to identify any deficiencies in the treatment, based on the degree of improvement or worsening of pain. The VAS can be used at the beginning and end of each appointment, recording the result in the progress report.

In the research, we will use the VAS to assess the vaginal complaints of the participants. They will receive an image of a ruler with markings from 0 to 10, where one end indicates the complete absence of symptoms, and the other indicates the worst possible symptom. For analysis, the Visual and Analog Scale of GSM symptoms (VAS - GSM) will classify symptoms from mild to severe based on the sum of one to twelve points ^[[88]](#endnote-88)^. This assessment will be applied to each of the symptoms: dyspareunia, dryness, irritation/burning, itching, and cytorrhagia, as shown in the table 3 below:

**Table 3 – Visual Analog Scale (VAS)**

| **VISUAL ANALOG SCALE (VAS)** | | | | |
| --- | --- | --- | --- | --- |
| **VAGINAL COMPLAINTS** | | 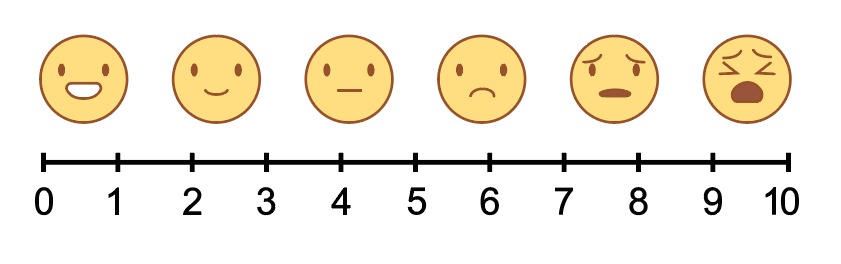  **Source: Own** | | |
| VAGINAL DRYNESS | |  |  |  |
| BURNING/IRRITATION | |  |  |  |
|  |  |  |  |  |
| DYSPAREUNIA (pain during sexual ntercourse) | |  |  |  |
|  |  |  |  |  |
| ITCHING | |  |  |  |
|  |  |  |  |  |
| COITAL BLEEDING (bleeding during sexual intercourse) | |  |  |  |
| ZERO = NO PAIN | | 1 - 3 = MILD | 4 - 7 = MODERATE | 8 - 10 = INTENSE |

**Source: Own**

The pelvic floor (PF) acts as support to the abdominal viscera during an increase in intra-abdominal pressure that can occur during a sneeze, cough, and other types of physical efforts. It is responsible for the mechanism of continence of the anal, urethral, and vaginal orifices in women ^[[89]](#endnote-89)^. The muscles involved in this structure are called pelvic floor muscles (PFM). The PFM are organized in layers and form a network of muscles that surround the urethral canal, vaginal canal, and anal canal, with the levator ani muscle being the main muscle involved in continence ^91^.

Due to the high prevalence of UI, various tools are used for the functional assessment of PFM, including digital palpation (DP) (subjective assessment), perineometry, electromyography (EMG), and dynamometry, which are considered objective assessments^[[90]](#endnote-90)^.

The dynamometer is a direct force assessment tool adapted to evaluate the contraction of the PFM, measuring in force units such as Newton, kilogram-force, or pound-force. The assessed force is the result of the maximum contraction of the PFM, and this same force is what leads to the closure of the urethral, vaginal, and anal canals during the elevation of intra-abdominal pressure, maintaining continence. Thus force is a fundamental parameter to be measured for the functional assessment of the PFM^91^.

A biofeedback device will be used to capture pressure in millimeters of mercury (mmHg) through a vaginal probe that is inflated with air. The biofeedback can be visualized both analogically using a manometer.

For the introduction of the vaginal probe, it will be covered with a non-lubricated male condom, as it is not possible to sterilize it. If necessary, a water-based lubricant gel will be applied to the external part of the male condom to facilitate the insertion of the probe. The pressure sensor will be inserted into the vaginal canal, and the resting vaginal pressure (muscles at rest) will be quantified. The device will be calibrated, zeroed, and the participant will be asked to contract her pelvic floor muscles inward and upward as strongly as possible three times and maintain the contraction for at least five seconds (maximum vaginal pressure), with a 30-second interval between each contraction ^[[91]](#endnote-91)^.

The assessment of the pelvic floor muscles will be conducted with the participant in a gynecological position, with the abdominal, pelvic floor, and leg regions exposed. This position provides a more accurate evaluation of isolated muscle contraction^[[92]](#endnote-92)^. The strength of the pelvic floor will be assessed before each laser application.

The Pad Test, or absorbent test, is a simple, easy, non-invasive, and effective method for evaluating urinary loss. The application of the Pad Test allows us to classify Stress Urinary Incontinence (SUI) as mild, moderate, and severe, based on the quantification of urine loss through the weighing of the sanitary pad ^[[93]](#endnote-93)^.

The technique does not necessarily reveal the total daily urinary loss of the patient; however, it allows quantifying the resulting Stress Urinary Incontinence (SUI) from common provocative exercises, simulating the daily routine of women. The one-hour Pad Test, validated by the Standardization Committee of the International Continence Society (ICS) in 1988, presents the advantages of speed and ease of execution, low cost, high specificity, and sensitivity when compared to urodynamic examination. It also provides an objective evaluation of the patient's complaints by a specialized professional, clarifying not only the technique but also addressing important and more precise clinical questions ^[[94]](#endnote-94)^.

Considering the high frequency of Stress Urinary Incontinence (SUI) in postmenopausal women and the fact that available diagnostic exams (excretory urography) are not always accessible in the public health system, we will use this resource for this research. The participant will receive a "Pad Test Kit" with an informative brochure (APPENDIX C) to perform it on the scheduled day.

The Pad Test procedure will be carried out as follows: Initially, the participant will be asked to place a pre-weighed pad near the external urethral meatus. Next, she will be given a 500 mL bottle of water to drink and instructed to rest for 15 minutes. Then, the participant will be asked to perform several actions simulating daily life activities (going up and down stairs for 15 minutes, sitting and standing ten times, coughing ten times, picking up objects from the floor five times, running in place for one minute, and washing hands under running water for one minute).

After completing the proposed activities, the pad will be removed and weighed on a precision scale with a minimum reading of 0.1 mg and a maximum capacity of 220 g. Urinary losses are assessed and classified as follows: losses up to 1 g are considered insignificant; between 1.1 and 9.9 g, they are classified as mild losses; between 10 and 49.9 g, they are moderate losses; and above 50 g, they are severe losses^[[95]](#endnote-95)^. The Pad Test will be performed before the first session and after the last laser intervention.

The vulvar temperature, in degrees Celsius, will be assessed at eight radiation points in the vulvar region (Figure 8) using a digital infrared thermometer, allowing non-contact measurement. The average of the eight readings will be calculated before and after each diode laser procedure in the four consecutive weekly sessions. Vaginal temperature will be evaluated before and after laser intervention in all applications.

**
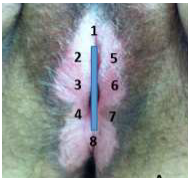
**

**Figure 8 – Vulvar Temperature Checkpoints**

**Sourrce: Belloto^39^**

**5.5. Expected Benefits:**

There is no direct benefit to the research participant.

## 5.6. Risks:

Possible risks involved, as found in the literature, are related to potential embarrassment when answering the FSI and ICIQ-SF questionnaires. There is also a possibility of late vaginal bleeding and/or discomfort during the application of the laser device in the vagina (external) and during the placement of the vaginal dynamometer due to the narrowing of the vaginal canal caused by menopausal symptoms. In case of any adverse event/complication, the participant will be supported and referred for medical evaluation (gynecologist at the unit) and nursing consultation by the Nurse at the unit (in this case, the researcher working at the research site).

**5.7. Sample Calculation:**

The sample size was calculated based on the formula described in the article "Sample size calculation," published in 2010 by Prashant Kadam and Supriya Bhalerao. Considering a Type I error of 0.05 and a test power of 80%, the calculated sample size was 60 participants, with 30 in each group (A and B). Figure 9 shows an analysis of the test power as a function of the sample size.

**Figure 9 - Analysis of test power as a function of effect size and sample size**


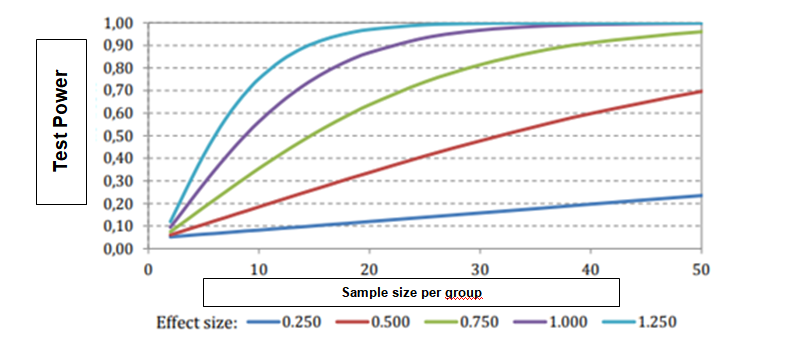


Figure 9 shows that, for medium (0.750) and large (1.000-1.250) effects, a minimum of 30 patients per group is sufficient to control the statistical variant, ensuring a power greater than 0.80. Considering a 10% loss of the sample, a total of 66 patients will be recruited for the research.

**5.8. Organization and Statistical Data Analysis**

The data will be tested for normality using the Shapiro-Wilk test, and if they exhibit parametric distribution, they will be represented by their respective means and standard deviations. If the assumption of parametric distribution is rejected, medians and quartiles will be used. To evaluate the association between the studied groups and independent variables, Student's t-test or Wilcoxon-Mann-Whitney test will be used for parametric and non-parametric data, respectively. All tests will be two-tailed, and the significance level adopted will be α = 0.05.

## 5.9. Randomization:

The data will be tested for normality using the Shapiro-Wilk test, and if they exhibit parametric distribution, they will be represented by their respective means and standard deviations. If the assumption of parametric distribution is rejected, medians and quartiles will be used. To evaluate the association between the studied groups and independent variables, Student's t-test or Wilcoxon-Mann-Whitney test will be used for parametric and non-parametric data, respectively. All tests will be two-tailed, and the significance level adopted will be α = 0.05.

.

### 5.9.1. Research Flowchart

###
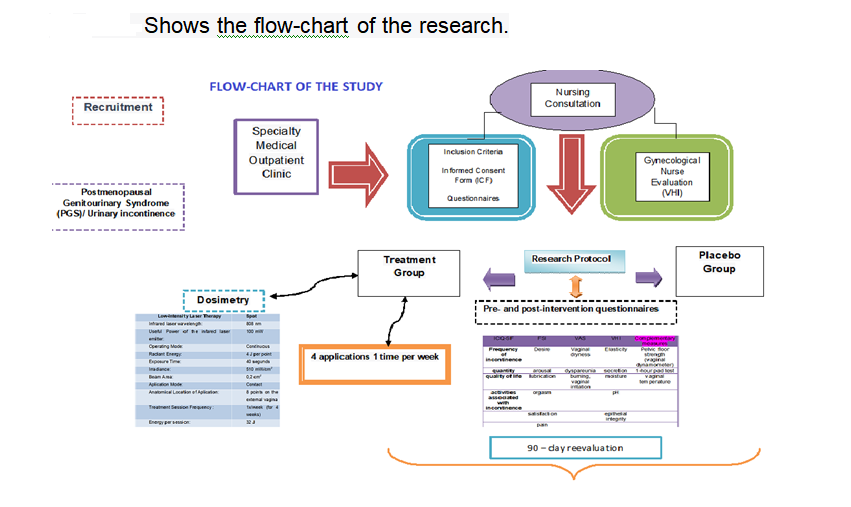


### 5.9. 2. Determination of groups and interventions:

The participants included in the study will be randomly divided into two groups: group A, which will receive photobiomodulation with external vaginal laser following the parameters according to Table 1, and group B (placebo) with the laser device turned off. Both treatments will be maintained for 4 consecutive weeks. The patient will be placed in a gynecological position, ensuring her privacy, and the 8 laser application points will be based on the Belotto protocol as per Table 4.

**Table 4 - Vaginal Laser Treatment Parameters**

| **Parameter** | **value** |
| --- | --- |
| Peak wavelength: | 808 nm |
| Operating Mode: | Continuous |
| Beam profile | Multimode |
| Polarization | random |
| Radiant power | 100 mW |
| Radiant Energy: | 4 J per point |
| Exposure duration: | 40 seconds |
| Beam Area at target: | 0.2 cm^2^ |
| Irradiance: | 500 mW/cm^2^ |
| Radiant exposure: | 20 J/cm² |
| Application Mode: | Close contact |
| Anatomical Location of Application: | 8 points on the external vagina |
| Treatment Sessions and Frequency: | Weekly, for 4 weeks |
| Total radiant energy per session: | 32 J |


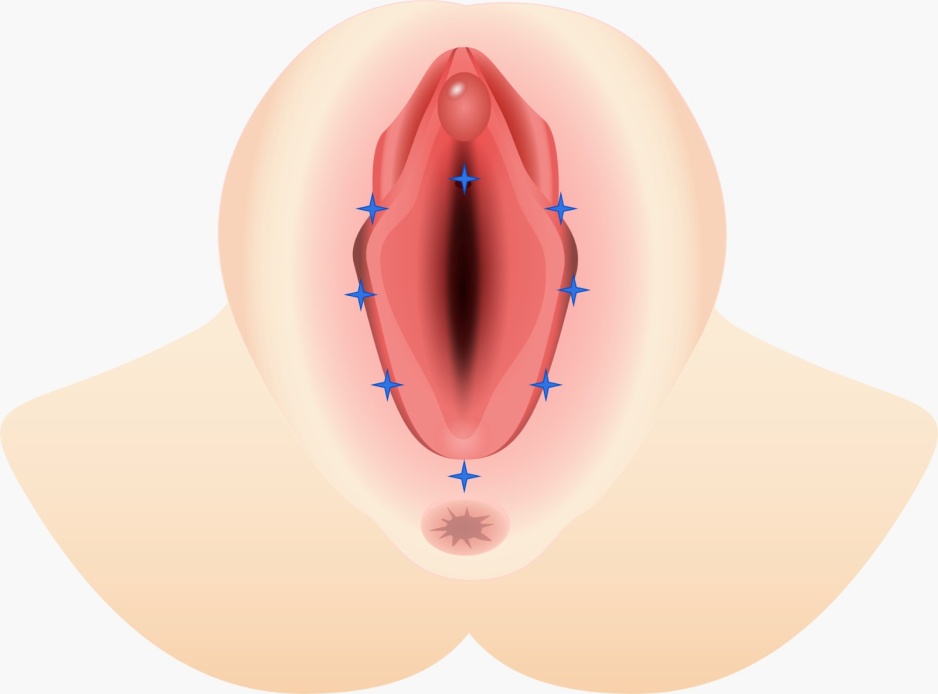


**Figure 10 - Vaginal Laser Application Points and Vulvar Temperature Check Locations.**

**SOURCE: own**

At admission, after the selection of participants, the procedures and evaluations described below will be carried out, which will be repeated in both groups at the end of treatment (1st follow-up), at 3 months (2nd follow-up), with the aim of assessing its immediate and short-term effects. After 6 months, contact will be made (by phone) with the participants to reassess complaints related to SGU and reapply the ICQ-SF and FSI questionnaires.

5.9.3. Intervention Procedure Steps:

**FIRST STAGE OF THE RESEARCH**

Researcher's Assessment: This stage will follow the following script:

1. An interview with the participant will be conducted to provide information about the research and obtain informed consent (APPENDIX I). All previously mentioned questionnaires will be administered.
2. The first questionnaire to be administered is the participant's sociodemographic questionnaire. After completing the questionnaire, the participant will receive a kit containing a bottle of water (500 ml) to start drinking and prepare for the 1-hour Ped Test, as well as a disposable pad (previously weighed) in a plastic bag.
3. The participant will be asked to go to the restroom, empty her bladder, put on the pad, and return to the room. The evaluation of sexual function (desire, arousal, lubrication, orgasm, satisfaction, and pain) will be conducted using the Female Sexual Function Index (FSFI) questionnaire.
4. The visual analog scale (VAS) will then be applied, questioning the intensity of all symptoms associated with SGU (dyspareunia, coital bleeding, dryness, irritation/burning, and vaginal itching).
5. While the participant drinks the 500 ml of water, the quality of life questionnaire on urinary incontinence (ICIQ-SF) will be administered, assessing the frequency of incontinence, quantity, social life discomfort, and activities associated with incontinence.
6. After the participant has consumed 500 ml of water and has been at rest for at least 15 minutes, she will be instructed on the Ped Test procedure, which will be performed for 1 hour as described earlier. This measurement will be repeated after the last treatment session and at the 90-day follow-up.
7. At this point, another evaluation will be conducted through a clinical interview form (APPENDIX E), developed by the researcher to obtain personal data and conduct a specific anamnesis. At the end, the participant will be released to go home with a scheduled return for the second stage of the research.

**SECOND STAGE OF THE RESEARCH**

1. An appointment with the unit's gynecologist will be scheduled for a blind pre-intervention assessment. During this appointment, a gynecological clinical examination of the participant will be conducted, applying the VHI (Vaginal Health Index) as described earlier. The gynecologist will assess elasticity, moisture, pH, epithelial integrity, and the amount of vaginal fluid, recording the findings on a specific form (APPENDIX F). After this evaluation, the patient will be referred/scheduled to start the proposed treatment of the research with the researcher.
2. On the day of the treatment (Treatment/placebo), the participant will be randomized as described earlier, then positioned in a gynecological position with her privacy preserved to start the protocol described in the flowchart below:


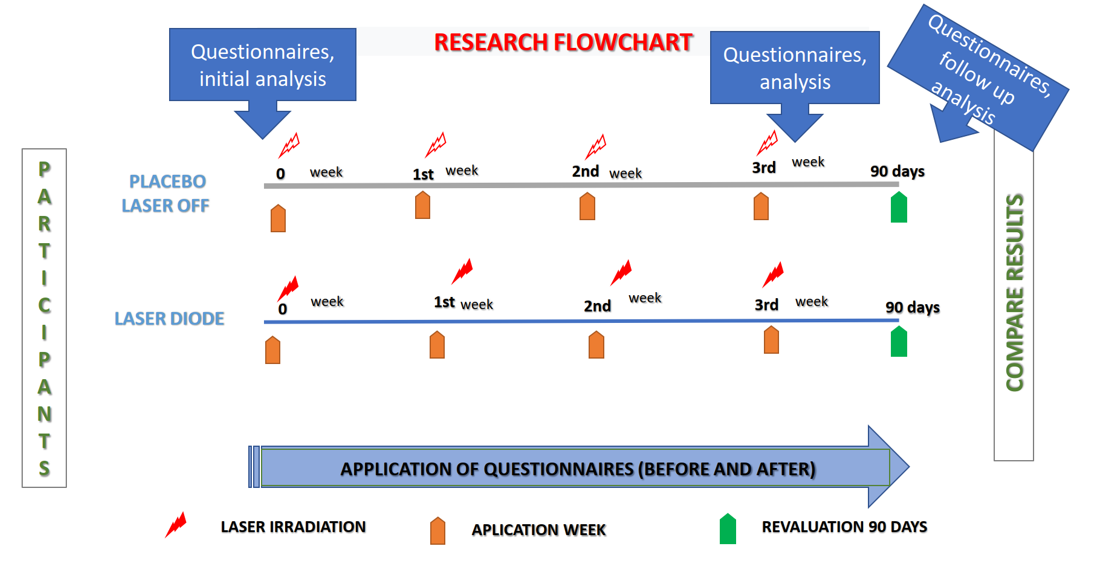


Figure 11 – Research Flowchart

**Source: author**

1. The vulvar temperature will be measured before and after each intervention week using a contactless infrared thermometer. Each measurement will require 30 seconds.
2. The next step is the measurement of pelvic floor muscle strength. The pressure sensor will be introduced into the participant's vaginal canal, where the resting vaginal pressure (muscles at rest) will be quantified. The device will be calibrated, zeroed, and the participant will be asked to contract their pelvic floor muscles as strongly as possible three times and maintain the contraction for at least five seconds (maximum vaginal pressure), with a 30-second interval between each pull. This measurement will be taken before laser interventions and after 90 days at the follow-up.
3. Upon leaving the office, the participant will receive a card with the next scheduled session. Each participant will have a follow-up form (pre- and post-intervention) with the information described in APPENDIX F.
4. At the end of the protocol, the participant's interest in continuing the research will be assessed to schedule a follow-up after 90 days with the gynecologist and researcher.
5. At the 90-day follow-up with the gynecologist, a blind gynecological clinical reassessment and reapplication of the VHI will be conducted for the doctor's opinion regarding the proposed treatment (treatment/placebo). After the medical consultation, the researcher will interview the participant, with a reapplication of the initial questionnaires and Ped-test, and the measurement of pelvic floor muscle strength (vaginal dynamometer), recording the information on a control form (APPENDIX G).
6. After 180 days from the start of the research protocol, the researcher will contact the participant by phone or in person for reassessment and reapplication of the FSIF, ICQ-SF, and VAS questionnaires.
7. If the study results indicate that laser (light) application brought improvements to the participants in the Treatment group (Group A), the treatment will also be made available to the placebo group (Group B) at the end of the study.

### 5.9.4. DATA ANALYSIS:

The data will be analyzed using SPSS software version 28. First, they will be tested for normality using the Shapiro-Wilk test, and if they exhibit a parametric distribution, they will be represented by their respective means and standard deviations. If the hypothesis of a parametric distribution is rejected, medians and quartiles will be used. Data with a parametric distribution will be subjected to Two-way ANOVA followed by the Student Newman-Keuls test for comparison between groups. The significance levels will be adjusted to 5% (p < 0.05).

**6. SCHEDULE:**

The planned schedule for the start of data collection will only be implemented "after approval from the Research Ethics Committee" through the CEP/CONEP System.

| Schedule |  |  |  |  |  |  |  |  |  |
| --- | --- | --- | --- | --- | --- | --- | --- | --- | --- |
|  | **-2** | **-1** |  | **0** | **1** | **2** | **3** | **4** | **5** |
| Literature Review and Project Update |  |  |  |  |  |  |  |  |  |
| Submit to COEP Uninove |  |  |  |  |  |  |  |  |  |
| Data Collection after COEP approval |  |  |  |  |  |  |  |  |  |
| Data Organization |  |  |  |  |  |  |  |  |  |
| Statistical Analysis |  |  |  |  |  |  |  |  |  |
| Qualification 2 |  |  |  |  |  |  |  |  |  |
| Project Update and Committee Corrections |  |  |  |  |  |  |  |  |  |

**7. FINAL CONSIDERATIONS**

Postmenopausal genitourinary syndrome (PGS) is associated with estrogen deficiency, leading to changes in the genitourinary organs of women that result in various urinary, genital, and sexual alterations. Vaginal atrophy, pain during sexual intercourse, and urinary loss are common complaints of menopausal women and have a significant impact on the quality of life of this population.

Laser therapy is a new practical and safe technology, painless, well-tolerated, and can be applied on an outpatient basis in the treatment of PGS. It has been considered an alternative treatment for women with atrophic vaginitis (AV) and a history of hormone-dependent neoplasms in which estrogen therapy may be contraindicated.

Given the high prevalence of PGS postmenopause and its impact on the overall health of women, along with the high costs of CO2 and Erbium lasers and the lack of specific protocols for treatment in this phase of life within public health systems, the study of new non-pharmacological therapies that can be used as adjuvant or substitute therapy is necessary.

Public health does not provide a specific care plan for this issue, nor does it offer hormone replacement medications and/or therapeutic alternatives for this population. The scientific community expects more studies to confirm the effects of the lasers currently used, to evaluate their effects on PGS, and to validate protocols. This study aims to contribute more results on the effectiveness of vaginal laser through photobiomodulation, pointing towards a non-hormonal proposal for the treatment of postmenopausal genitourinary syndrome with improvement in signs and symptoms and quality of life in this vital cycle of female senescence.

# REFERENCES:

1. 1. Portman DJ, Gass ML; Vulvovaginal Atrophy Terminology Consensus Conference Panel. Genitourinary syndrome of menopause: new terminology for vulvovaginal atrophy from the International Society for the Study of Women's Sexual Health and the North American Menopause Society. Menopause. 2014 Oct; [Acesso em 04/06/2021]; 21(10): 1063-8.

   [↑](#endnote-ref-1)
2. Brasil, Ministério da Saúde. Banco de dados do Sistema Único de Saúde-DATASUS. Availablefrom://tabnet.datasus.gov.br/cgi/tabcgi.exe?ibge/cnv/projpopbr.def. . [↑](#endnote-ref-2)
3. Andrade RL, Fernandes ACM, Dias JRP, Laurindo BM, Vieira RC. Evaluation of the quality of life of menopausal women attended in a specialized outpatient clinic. Brazilian Journal of health Review. 2019; 2(1): 66-90. [↑](#endnote-ref-3)
4. 1. Palacios S. Managing urogenital atrophy. Maturitas. 2009 Aug; 20; 63(4):315-8.
   2. Di Bonaventura M, Luo X, Moffatt M, Bushmakin AG, Kumar M, Bobula J. The Association Between Vulvovaginal Atrophy Symptoms and Quality of Life Among Postmenopausal Women in the United States and Western Europe. J Womens Health (Larchmt). 2015 Sep; 24 (9): 713-22.

   [↑](#endnote-ref-4)
5. [↑](#endnote-ref-5)
6. 1. Keil K. Urogenital atrof: diagnosis, consequences and management. Curr Womens Health Rep. 2002; 2: 305-311.

   [↑](#endnote-ref-6)
7. Behnia-Willison F, Sarraf S, Miller J, Mohamadi B, Care AS, Lam A, et al. Safety and long-term efficacy of fractional CO2 laser treatment in women suffering from genitourinary syndrome of menopause. Eur J Obstet Gynecol Reprod Biol. 2017; [acesso em 04/06/2021]; 213:39-44. [↑](#endnote-ref-7)
8. Argote OLA, Mejía RME, Vásquez TML, Villaquirán GME. Climaterio y menopausia en mujeres afrodescendientes: una aproximación al cuidado desde su cultura. Aquichán. 2008 [acesso em 05/06/2021];  8(1): 33-49. [↑](#endnote-ref-8)
9. 1. Pernas G A, Pérez PJ. Síndrome climatérico. Caracterización y factores de riesgo. Villa Clara. Rev. Temas Estadísticos de Salud. 2005.

   [↑](#endnote-ref-9)
10. Guarisi T, Pinto-Neto A, Osis MJ, Orcesi A, Costa-Paiva LHS, Faúndes A. Seeking medicalcare by women with urinary incontinence. Rev. Brás. Ginecol. Obstet. 2001. [acesso em 05/06/21]; 23: 439-443. [↑](#endnote-ref-10)
11. Valadares AL, Pinto-Neto AM, Conde DM, Osis MJ, Sousa MH, Costa-Paiva L. The Sexuality of middle-aged women with a sexual partner: a populationbased study. Menopause 2008; 15: 706-713. [↑](#endnote-ref-11)
12. Luiz filho JF, Baccaro LF, Fernandes T, Conde DM, Costa-Paiva, Pinto Neto AM. Factors associated with menopausal symptoms in women from a metropolitan region in Southeastern Brazil: a population-based household survey. Rev Bras Ginecol Obstet. 2015 [Acessado 05/06/21]; 37 (4): 152-158. [↑](#endnote-ref-12)
13. Lara LA, Useche B, Ferriani RA, Reis RM, de Sá MF, de Freitas MM, Rosa e Silva JC, Rosa e Silva AC. The effects of hypoestrogenism on the vaginal wall: Interference with the normal sexual response. J. Sex. Med. 2009 [acessado 05/06/21]; 6: 30 – 39. [↑](#endnote-ref-13)
14. Kingsberg AS, Wysocki S, Magnus L, Krychman ML. Vulvar and Vaginal Atrophy in Postmenopausal Women: Findings from the REVIVE (REal Women`s Views of Treatment Options for Menopausal Vaginal ChangEs ) Survey. J. Sex. Med 2013; 10: 1790-9. [↑](#endnote-ref-14)
15. MacBride MB, Rhodes DJ, Shuster LT. Vulvovaginal atrophy. Mayo Clin Proc 2010; 85:87–94. [↑](#endnote-ref-15)
16. Sinha A, Ewies AAA. Non-hormonal topical treatment of vulvovaginal atrophy: an up-to-date overview. Climacteric 2013; 16: 305–12.acessado 05/06/21 [↑](#endnote-ref-16)
17. The North American Menopause Society. Management of symptomatic vulvovaginal atrophy: 2013 position statement of The North American Menopause Society. Menopause (New York, NY). 2013; 20(9): 888-902; quiz 3-4. acessado 05/06/21. [↑](#endnote-ref-17)
18. Salvatore S, Nappi RE, Zerbinati N, Calligaro A, Ferrero S, Origoni M, et al, A 12-week treatment with fractional CO2 laser for vulvovaginal atrophy: a pilot study. Climateric 2014[Acesso em 05/06/21]; 17:363–9. [↑](#endnote-ref-18)
19. Gaspar A, Brandi H, Gomez V, Luque D. Efficacy of Erbium: YAG laser treatment compared to topical estriol treatment for symptoms of genitourinary syndrome of menopause. Lasers in surgery and medicine. 2017; 49(2): 160-8. [↑](#endnote-ref-19)
20. Gaspar A, Maestri S, Silva J, et al. Intraurethral Erbium: YAG laser for the management of urinary symptoms of genitourinary syndrome of menopause: A pilot study. Lasers in surgery and medicine. 2018;50(8):802-7. [↑](#endnote-ref-20)
21. Nilsson K, Risberg B, Heimer G. The vaginal epithelium in the postmenopausecytology, histology and pH as methods of assessment. Maturitas. 1995; 21 (1): 51-56. [↑](#endnote-ref-21)
22. ALR Pereira. Laser treatment in atrofic vaginitis. Master’s thesis in Mediciane. University of Porto. 2019. [↑](#endnote-ref-22)
23. Gambacciani M, Palacios S. Laser therapy for the restoration of vaginal function. Maturitas. 2017; 99: 10-15.. [↑](#endnote-ref-23)
24. 1. Laryssa Caroline Torres Severiano, Kayssa Ferreira Pena, Giulia Victorino Miranda, Cláudia Teixeira da Costa Lodi. Laser therapy in the treatment of Genitourinary Syndrome of Postmenopause. E-Scientia. 2019;  [12( 2)](https://revistas.unibh.br/dcbas/issue/view/156).

    [↑](#endnote-ref-24)
25. Montenegro CABR. Obstetrícia Fundamental. 12^a^ edição. Rio de Janeiro. Guanabara Koogan, 2011. [↑](#endnote-ref-25)
26. Heitmann RJ. Anatomy of the Female Genital System. In: CURRENT Diagnosis and Treatment in Gynecology and Obstetrics. 11th. ed. Porto Alegre: AMGH, 2014. p. 36–37 [↑](#endnote-ref-26)
27. Gomes E F. Histologia e Citologia do Aparelho Genital Feminino. Histologia e Citologia do Aparelho Genital Feminino. [↑](#endnote-ref-27)
28. Junqueira LC, Carneiro J. Basic Histology. 12 th ed. Rio de Janeiro: Guanabara Koogan, 2013. v. 1 [↑](#endnote-ref-28)
29. De Landsheere L, et al. Histology of the vaginal wall in women with pelvic organ prolapse: a literature review. International urogynecology journal. [Acesso em 17/06/21]; 24(12): 2011–2020. [↑](#endnote-ref-29)
30. Morgan-Martins MI, Jacques SI, Hartman RM, Marques C, Marroni CA, Marroni NP. Protection of estrogen in portal hypertensiongastropaty: an experimental model. Arq Gastroenterol. 2011; 48: 211-216. [↑](#endnote-ref-30)
31. Aldrighi JM, Aldrighi CMS, Aldrigh APS. Systemic changes in menopause. Rev Bras Méd. 2002;15:21. [↑](#endnote-ref-31)
32. Pereira PWQS, Corrêa FJS, Brasileiro JPB. Manual of Gynecology from The Brasilia Society of Gynecolgy and Obstetrics. Brasília: Luan Communication Editor. 2017 [↑](#endnote-ref-32)
33. Baracat EC, Haidar M, Nunes MG, et al. IN: Ambulatory and Hospital Medicine Guides. UNIFESP/ Escola Paulista de Medicina. Barueri, SP: Manole, 2005 [↑](#endnote-ref-33)
34. Ferreira VN, Chinelato RSC, Castro MR, Ferreira MEC. Menopause: Biopsychosocial Framework for Female Aging. Psicol. Soci. 2013; 25(2): 410- 419. [↑](#endnote-ref-34)
35. Antunes S, Marcelino O, Aguiar T. Pathophysiology of menopause. Ver. Port. Clin. Geral 2003; 19:353-76. [↑](#endnote-ref-35)
36. Leal JWB, Ribeiro CBL. Pathophysiology of pre-menopause. Moreira Jr. Editora. 2014. [↑](#endnote-ref-36)
37. Graef AM, Locatelli C, Santos P. Use of soy (Glycine Max) and Angelica sinensis (Dong Quai) phytoestrogens as a therapeutic alternative for treating climateric symptoms. Evidência. 2012; 12(1): 83-96.   [↑](#endnote-ref-37)
38. [Avis NE, Crawford SL, Greendale G, et al](https://www.ncbi.nlm.nih.gov/pubmed/25686030). Duration of menopausal vasomotor symptoms over the menopause transition (Study of Women's Health Across the Nation). JAMA Intern. Med. 2015 [acesso em 17/06/2021]; 175 (4):531–539. [↑](#endnote-ref-38)
39. SOBRAC ( Brazilian Society of Climacteric). Brazilian Multidisciplinary Consensus on Healthcare for Climateric Women. 2003 [↑](#endnote-ref-39)
40. Selbac MT, Fernandes CGC, Marrone LCP, Vieira AG, Silveira, Morgan- Martins MI. Behavioral and physiological changes determined by the female biological cycle – climateric to menopause. Aletheia. 2018; 51(12): 177-190. [↑](#endnote-ref-40)
41. Boucinha MST. Associations between serum levels of growth factors, insulin, and leptin with prognostic factors of breast cancer. [Dissertation]. Porto Alegre: Federal University of Rio Grande do Sul (UFRGS). 2012. [↑](#endnote-ref-41)
42. Pedrosa DF, Rezende LCD, Silva IV, Rangel LBA, Gonçalves WLS, Graceli JB. The beneficial effects of estrogen on the cardiovascular system. Perspectives. 2009; 3(12), 190-196. [↑](#endnote-ref-42)
43. 1. Kannel WB, et al. Menopause and risk of cardiovascular disease. The Framinghan study.Annals of Internal Medicine. 1976; 85(4): 447– 452.

    [↑](#endnote-ref-43)
44. Biglia N, et al. Vasomotor symptoms in menopause: a biomarker of cardiovascular disease risk and other chronic diseases? Climacteric. 2017; 20(4): 306-312. [↑](#endnote-ref-44)
45. Antunes S, Marcelino O, Aguiar T. The physiopathology of menopause. The Climateric and menopause Dossier. Ver. Port. Clin. Geral. 2003; 19:353-7. [↑](#endnote-ref-45)
46. Amadei SU, Silveira VAS, Pereira AC, Carvalho YR, Rocha RF. The influence of estrogen deficiency on bone remodeling and repair process. J. Bras. Patol. Med. Lab. 2006; 42(1): 5-12. [↑](#endnote-ref-46)
47. Compston JE. Sex steroids and bone. Physiological Reviews. 2001; 81(1): 419- 47. [↑](#endnote-ref-47)
48. Pinkas J, et al. The polymorphism of estrogen receptor α is important for metabolic consequences associated with menopause. Endokryno.l Pol. 2016; 67(6): 608-614. [↑](#endnote-ref-48)
49. Kamilos, MF, Borrelli CL. New therapeutic option in genitourinary syndrome of menopause: pilot study using microablative fractional radiofrequency. Einstein (São Paulo). 2017 [Acesso em 28/08/ 2021]; 15(4): 445-451. [↑](#endnote-ref-49)
50. Hodgins MB, Spike RC, Mackie RM, MacLean AB. An immunohistochemical study of androgen, oestrogen and progesterone receptors in the vulva and vagina. BJOG: An International Journal of Obstetrics and Gynaecology. 1998; 105(2): 216–222.  [↑](#endnote-ref-50)
51. Cavada LF, Nunes A, Pinheiro M, Silva PT. Approaches to menopause in primary health care. Acta Med Port.2010. [↑](#endnote-ref-51)
52. Society of Obstetricians and Gynaecologists of Canada: Canadian Consensus Conference on Menopause Update. J. Obstet. Gynaecol.2009;S1-S48. [↑](#endnote-ref-52)
53. 1. Brazil. Ministry of Health. Secretariat of Health Care. Department of Strategic Programmatic Actions. Women's Care Manual in Climacteric/Menopause / Ministry of Health, Secretariat of Health Care, Department of Strategic Programmatic Actions. – Brasília : Ministry of Health Publisher, 2008.

    [↑](#endnote-ref-53)
54. Soules MR, Sherman S, Parrot E, Rebar R, Santoro N, Utian W, ET AL. Executive Sumary: Estages Of Reproductive Aging Workshop (STRAW). Fertility and Sterility. 2001; 76(5): 874-78. [↑](#endnote-ref-54)
55. Pompei LM, Machado RB, Wender COM, Fernandes CE. Brazilian Consensus on Hormone Therapy for Menopause – Brazilian Climateric Associatio (SOBRAC). São Paulo. Leitura Médica, 2018. [↑](#endnote-ref-55)
56. 1. Bezerra TA, Lima ECS, Araújo AL, Rosário KD. Hormone Replacement Therapy in menopause. Rev Inic Cient Ext. 2019; 2(4):247-9.

    [↑](#endnote-ref-56)
57. Wannmacher L, Lubianca JN. Hormone Replacement Therapy in menopause: Current evidence. Brasília; 2004; 1(6). [↑](#endnote-ref-57)
58. Polonini HC. Hormone Replacement and Women’s Health in Climateric: risks and benefits. Rev. APS. 2011; 14(3):354-61 [↑](#endnote-ref-58)
59. Padini D. Hormone Replacement Therapy. Arq. Bras. Endocrinol. Metab. 2014; 58:2 [↑](#endnote-ref-59)
60. Fernandes TR. Vaginal treatment for genitourinary syndrome of menopause: a randomized controlled trial. 2018. [↑](#endnote-ref-60)
61. Nachtigall LE. Comparative study: Replens versus local estrogen in menopausal women. Fertility and Sterility 1994, 61: 178-180. [↑](#endnote-ref-61)
62. Bygderman M, Swahn ML. Polycarbophil Acid versus dienoestrol cream in the symptomatic treatment of vaginal atrophy in postmenopausal women. Maturitas 1996; 23 (3): 259 – 263 [↑](#endnote-ref-62)
63. Mendes MC, Lara LA, Sá MF. Síndrome geniturinária da menopausa. Femina. 2020;48(4):198-207 [↑](#endnote-ref-63)
64. Goldstein SR, Bachmann GA, Koninckx PR, Lin VH, Portman DJ, Ylikorkala O, et al. Ospemifene 12-month safety and efficacy in postmenopausal women with vulvar and vaginal atrophy. Climacteric. 2014;17(2):173-82 [↑](#endnote-ref-64)
65. Wańczyk-baszak J, Woźniak S, Milejski B, Paszkowski T. Genitourinary syndrome of menopause treatment using lasers and temperature-controlled radiofrequency. 2018;17(4):180–4. [↑](#endnote-ref-65)
66. Cavalcanti TM, et al. Knowledge of the physical properties and interaction of laser with biological tissue in dentistry. Anais Brasileiros de Dermatologia. 2011[acesso em 04/09/2021]; 86 (5):955–960. [↑](#endnote-ref-66)
67. Ronconi L, Galli , MonaLisa TTM. The latest frontier in the treatment of vaginal atrophy. [s.l.] DEKA ed. 2012; 2. [↑](#endnote-ref-67)
68. Tadir Y, et al. Light and energy based therapeutics for genitourinary syndrome of menopause: Consensus and controversies. Lasers in Surgery and Medicine. 2017; 49(2): 137–159. [↑](#endnote-ref-68)
69. Zezell DM. Interaction of Laser Light. Handbook of the Professional Master’s Degree Course in Lasers in Dentistry. 2005; IPEN/FOUSP: 24R30. [↑](#endnote-ref-69)
70. Silva KSBS. Efficacy of vaginal CO2 laser in the treatment of genitourinary syndrome of menopause compared to the use of topical promestriene: a clínical study. [Thesis]. Porto alegre: Federal University of Rio Grande do Sul (UFRGS).2020. [↑](#endnote-ref-70)
71. Gambacciani M, Palacios S. Laser therapy for the restoration of vaginal function. Maurita. 2017; 99:10 – 15. [↑](#endnote-ref-71)
72. Wallace SL, St Martin B, Lee K, Sokol ER. A cost-effectiveness analysis of vaginal carbon dioxide laser therapy compared with standard medical therapies for genitourinary syndrome of menopause-associated dyspareunia. Am J Obstet Gynecol. 2020; 223(6): 890. [↑](#endnote-ref-72)
73. Pieralli A, Bianchi C, Longinotti M, Corioni S, Auzzi N, Becorpi A, et al. Long ‑ term reliability of fractioned - CO2 laser as a treatment for vulvovaginal atrophy (VVA) symptoms. Arch Gynecol Obstet. 2017*.*  [↑](#endnote-ref-73)
74. Ahluwalia J, Avram MM, Ortiz AE. Lasers and energy-based devices marketed for vaginal rejuvenation: A cross-sectional analysis of the MAUDE database. Lasers Surg Med. 2019; 51(8):671-677. [↑](#endnote-ref-74)
75. Alshiek et AL. Vaginal Energy-Based Devices. Female Pelvic Medicine & Reconstructive Surgery. 2020; 26(5). [↑](#endnote-ref-75)
76. Haelle T. Lasers Promising for Genitourinary Syndrome of Menopause. 2018; [↑](#endnote-ref-76)
77. Filippini M, et al. Efficacy of fractional CO2 laser treatment in postmenopausal women with genitourinary syndrome: a multicenter study. Menopause. 2020[acessado em 24/09/2021]; 27(1): 43 - 49. [↑](#endnote-ref-77)
78. Henstschel H, et al. Validação do Female Sexual Fuction Index (FSFI) para uso na língua portuguesa. Revista do HCPA. 2007; 27(1): 12. [↑](#endnote-ref-78)
79. Wiegel M, Meston C, Rosen R. The Female Sexual Function Index (FSFI): cross-validation and development of clinical cutoff scores. J. Sex. Marital Ther. 2005; 31:1-20.    [↑](#endnote-ref-79)
80. Avery K, Donovan J, Abrams P. Validation of a new questionnaire for incontinence: the International Consultation on Incontinence Questionnaire (ICIQ). Abstract nº 86 of the International Continence Society 31st annual meeting. Seoul, Korea. Neurourol Urodynamics 2001; 20:510-1. [↑](#endnote-ref-80)
81. Tamanini JTN, et al. Validation of the “International Consultation on Incontinence Questionnaire - Short Form” (ICIQ-SF) for Portuguese. Revista de Saúde Pública. 2004; 38( 3): 438 – 444. [↑](#endnote-ref-81)
82. 1. Rosa LHT, Souza CM, Lima CHL, Boggio ESB, Santos FC, Carboni C, et al. Prevalência de incontinência urinária em idosos de Porto Alegre-RS. Geriatr. Gerontol. Aging. 2014; 8: 104-109.

    [↑](#endnote-ref-82)
83. Sjöström M, Umefjord G, Stenlund H, Carlbring P, Andersson G, Samuelsson E. Internet-based treatment of stress urinary incontinence: a randomised controlled study with focus on pelvic floor muscle training. BJU Int. 2013; 112(3): 362-72. [↑](#endnote-ref-83)
84. Gloria Bachmann,Urogenital ageing: an old problem newly recognized. Maturitas. 1995 [acesso em 26/09/2021]; 22(1 supplement ): S1-5. [↑](#endnote-ref-84)
85. Roy S, et al. Vaginal pH is similar to follicle-stimulating hormone for menopause diagnosis. American Journal of Obstetrics and Gynecolog. 2004; 190(5): 1272–1277. [↑](#endnote-ref-85)
86. Caillouette JC, Sharp CF Jr, Zimmerman GJ, Roy S. Vaginal pH as a marker for bacterial pathogens and menopausal status. Am J Obstet Gynecol. 1997; 176(6): 1270-5; discussion 1275-7. [↑](#endnote-ref-86)
87. Linhares IM, Summers PR, Larsen B, Giraldo PC, Witkin SS. Comtemporary Perspectives on vaginal pH and lactobacilli. American Journal of obstetrics and Gynecology. 2011; 204 (2): 120 e 1. [↑](#endnote-ref-87)
88. Magon N, Alinsod R, Thermi V.The Revolutionary Technology for Vulvovaginal Rejuvenation and Noninvasive Management of Female SUI. J. Obstet. Gynaecol. India. 2016; 66 (4): 300-2. [↑](#endnote-ref-88)
89. Mittal R, Liu J, Nager C. Pelvic floor function diagnostic and therapeutic station and uses thereof. US.2007. [↑](#endnote-ref-89)
90. 1. Orth DL. Methods of direct assessment of the strength of female pelvic floor muscles: systematic review and utility model. [Thesis]. São Carlos: University of São Carlos. 2016.

    [↑](#endnote-ref-90)
91. Barbosa PB, Franco MM, Souza Fde O, Antônio FI, Montezuma T, Ferreira CH. Comparison between measurements obtained with three different perineometers. Clinics. São Paulo. 2009; 64(6): 527-33. [↑](#endnote-ref-91)
92. Bø K, Finckenhagen HB. Is there any difference in measurement of pelvic floor muscle strength in supine and standing position? Acta. Obstet. Gynecol. Scand. 2003; 82(12): 1120-4. [↑](#endnote-ref-92)
93. Abrams P, Cardozo L, Fall M, Griffiths D, Rosier P, Ulmsten U, et al. The standardisation of terminology in lower urinary tract function: report from the standardisation sub-committee of the International Continence Society. Urology. 2003; 61(1): 37- 49. [↑](#endnote-ref-93)
94. Yang JM, Yang SH, Yang SY, Yang E, Huang WC, Tzeng CR. Clinical and pathophysiological correlates of the symptom severity of stress urinary incontinence. Int Urogynecol J Pelvic Floor Dysfunct. 2010; 21(6): 637-43. [↑](#endnote-ref-94)
95. Albuquerque, Maria Thereza et al. Correlation between complaints of stress urinary incontinence and the one-hour pad test postmenopausal women. Brazilian Journal of Gynecology and Obstetrics. 2011; 33(2): 70-74. [↑](#endnote-ref-95)
